# Selective recruitment of stress-responsive mRNAs to ribosomes for translation by acetylated protein S1 during nutrient stress in *Escherichia coli*

**DOI:** 10.1038/s42003-022-03853-4

**Published:** 2022-09-01

**Authors:** Bai-Qing Zhang, Zong-Qin Chen, Yu-Qi Dong, Di You, Ying Zhou, Bang-Ce Ye

**Affiliations:** 1 Laboratory of Biosystems and Microanalysis, State Key Laboratory of Bioreactor Engineering, East China University of Science and Technology, Shanghai, 200237 China; 2 Institute of Engineering Biology and Health, Collaborative Innovation Center of Yangtze River Delta Region Green Pharmaceuticals, College of Pharmaceutical Sciences, Zhejiang University of Technology, Hangzhou, 310014 Zhejiang China

**Keywords:** Cellular microbiology, Acetylation

## Abstract

The chemical modification of ribosomes plays an important regulatory role in cellular translation adaptation in response to environmental stresses. Nevertheless, how the modified ribosome reprograms the translation machinery for the preferential expression of the specific mRNAs encoding stress-responsive proteins to stress remains poorly understood. Here, we find that AcP-induced acetylation of K411 and K464 in ribosomal protein S1 during carbon-nitrogen imbalance, which in turn impacts its binding with distinct mRNAs. S1 acetylation shows differential selectivity for recruiting subsets of mRNAs to ribosomes. Using the RNC-Seq method, we find that mimic acetylated S1 prefers transcripts related with the formation of flagella/biofilms, two-component systems, nitrogen assimilation, amino acid degradation, and lipopolysaccharide biosynthesis, whereas inhibits the translation of mRNAs involved in amino acid biosynthesis and most ribosomal proteins. Importantly, further characterization of S1-binding site (SBS) sequences of mRNAs with different translation efficiencies indicated that the presence of a conserved motif allows coordinated regulation of S1 acetylation-driven translation reprogramming for cell survival during nitrogen starvation. These findings expand the repertoire of ribosome heterogeneity to the acetylation level of S1 at specific sites and its role in the ribosome-mediated regulation of gene expression as a cellular response at the translational level to stress.

## Abstract (web summary)

RNA molecular chaperone S1 is acetylated and selectively recruits stress-responsive mRNAs to the ribosome during nitrogen starvation in *E. coli*, revealing a translation regulation mechanism for nutrient stress adaptation.

## Introduction

Throughout their life, bacteria face different types of unpredictable environmental stress and challenges. To grow and survive, bacterial cells must rapidly induce dramatic cellular reprogramming at both the transcriptional and translational levels to respond to and cope with these stresses (particularly under nutrient deficiency). The molecular mechanisms of transcriptional regulation in response to different environmental stresses have been widely investigated in the model bacterium *Escherichia coli* over the past several decades. It was revealed that a highly integrated transcriptional regulatory network with several sigma factors of RNA polymerase, two-component systems, and small signaling molecules (such as cAMP, (p)ppGpp, and c-di-GMP) evolved to detect and adapt to environmental changes^1,2^. Recent studies have also shown a lack of correlation between the mRNA and protein levels of numerous genes^3,4^ and have suggested that regulation at the level of protein synthesis provides bacteria with plasticity to have rapid and fine-tuned responses to nutrient availability as well as to physical, chemical, and biological stresses^5,6^. However, the underlying molecular mechanism permitting rapid and selective changes in the proteome landscape upon translational control in response to nutrient status and environmental cues in *E. coli* remains poorly understood.

Translational regulation plays a crucial role in cell growth, survival, stress response, and environmental adaptation, particularly under nutrient deficiency^7–9^. The ribosome is the protein-synthesizing machine in the cell and therefore lies at the heart of translational regulation for determining the proteome landscape of the cell for stress adaptation. It is becoming increasingly evident that the heterogeneity of translational machinery with selective translation allows cells to rapidly respond to a variety of stress conditions^10,11^. The heterogeneity of the translational apparatus includes differences in the composition of ribosomal proteins (RPs)^12^, ribosomal RNA (rRNA) diversity, chemical modification of RPs and rRNAs^13–15^, and the activity of ribosome-associated factors^8,16^. Bacteria can reprogram the translation machinery (translational reprogramming) to rapidly fine-tune the levels of specific protein products using specific ribosomes resulting from heterogeneity. The chemical modification (acetylation, methylation, phosphorylation, ubiquitylation, and O-GlcNAcylation) of RPs, translation factors, and ribosome-associated proteins, as an important kind of heterogeneity of the translational apparatus, has been increasingly discovered^15–18^, although the majority of these have yet to be functionally characterized.

Translation (protein synthesis) consumes more than half of the energy and cellular resources^19^. To save cellular energy and resources, translation must be tightly coupled to nutrient availability and strictly regulated at the point of initiation. The recruitment of mRNAs is the key regulatory step of translation initiation. It is an economic and competitive strategy in which only specific proteins are expressed by selective recruitment and initiation of stress-responsive mRNAs using specific modified translation initiation factors. Some nutrient signaling pathways and molecular mechanisms based on the chemical modification of eukaryotic initiation factors (eIFs) have been revealed to lead to either global or transcript-specific control of translation initiation. Amino acid depletion leads to phosphorylation of eIF2α at serine residue 51, which inhibits ternary complex formation and general protein synthesis^20^. The eIF4F complex consists of eIF4E, eIF4G, and eIF4A, of which eIF4A plays an important role in promoting recruitment of mRNAs to ribosomes^21,22^. CDKA-mediated phosphorylation (Thr-164) of eIF4A results in a decrease in eIF4A activity, thereby inhibiting protein translation^23^. The nutrient-sensing mTORC1-regulated phosphorylation of 4EBP1 (eIF4E–binding proteins) controls its binding and dissociation with eIF4E for translation of selective mRNAs: free eIF4E promotes cap-dependent translation^24^, while binding of eIF4E with 4EBP1 significantly affects some mRNAs containing a 5′-terminal oligopyrimidine tract^25^. Recent work found that CK2-mediated phosphorylation of eIF3d inhibits its cap-binding activity and tunes the translation of some mRNAs involved in glucose homeostasis. The results demonstrated that eIF3d-directed translation adaptation based on CK2-mediated phosphorylation was essential for cell survival during chronic glucose deprivation^13^.

In gram-negative bacteria, mRNA recruitment to ribosomes through the binding of protein S1 is a critical step in the regulation of gene expression that impacts which mRNAs are translated. Protein S1 is necessary for most canonical mRNA translation initiation in *E. coli*^26^. It is composed of the N-terminal domain (NTD) involved in the assembly of S1 with ribosomes via S1-S2 interactions and the C-terminal domain (CTD) responsible for binding with mRNA, unfolding of secondary structures, and delivery of mRNA to ribosomes. Some chemical modifications (phosphorylation and acetylation) of S1 have been reported^27,28^. However, it remains largely unknown how these chemical modifications regulate selective mRNA translation during stress to allow cells to rapidly respond to a variety of stress conditions.

Here, we found that two key lysine sites (K411 and K464) in the CTD domain of RP S1 are acetylated by acetyl phosphate (AcP) during nitrogen starvation in *E. coli*, which in turn impacts its binding with distinct mRNAs, leading to selective recruitment of a subset of mRNAs to ribosomes for translation. We found that the S1K411/464Ac protein (designated “nitrogen starvation stress S1”) preferentially translates a set of transcripts enriched in the formation of flagella and biofilms, two-component systems, nitrogen assimilation, amino acid degradation, and lipopolysaccharide biosynthesis but inhibits the translation of some mRNAs involved in amino acid biosynthesis and most RPs. The results uncovered an uncharacterized stress adaptation mechanism based on acetylation of S1 for cell survival during nitrogen starvation. Altogether, a potential strategy for translational reprogramming during nutrient stress in bacteria is to selectively recruit different subsets of mRNAs (stress-responsive mRNAs) to ribosomes for translation through the chemical modification of protein S1. On the other hand, some specific motifs were identified and designed based on bioinformatics analysis of S1-binding site (SBS) sequences of mRNAs with different translation efficiencies and can be employed as biobricks of synthetic biology for fine-tuned gene expression.

## Results

### Acetylation of K411 and K464 in S1 induced by AcP influences its interaction with RNA

**Fig. 1:**
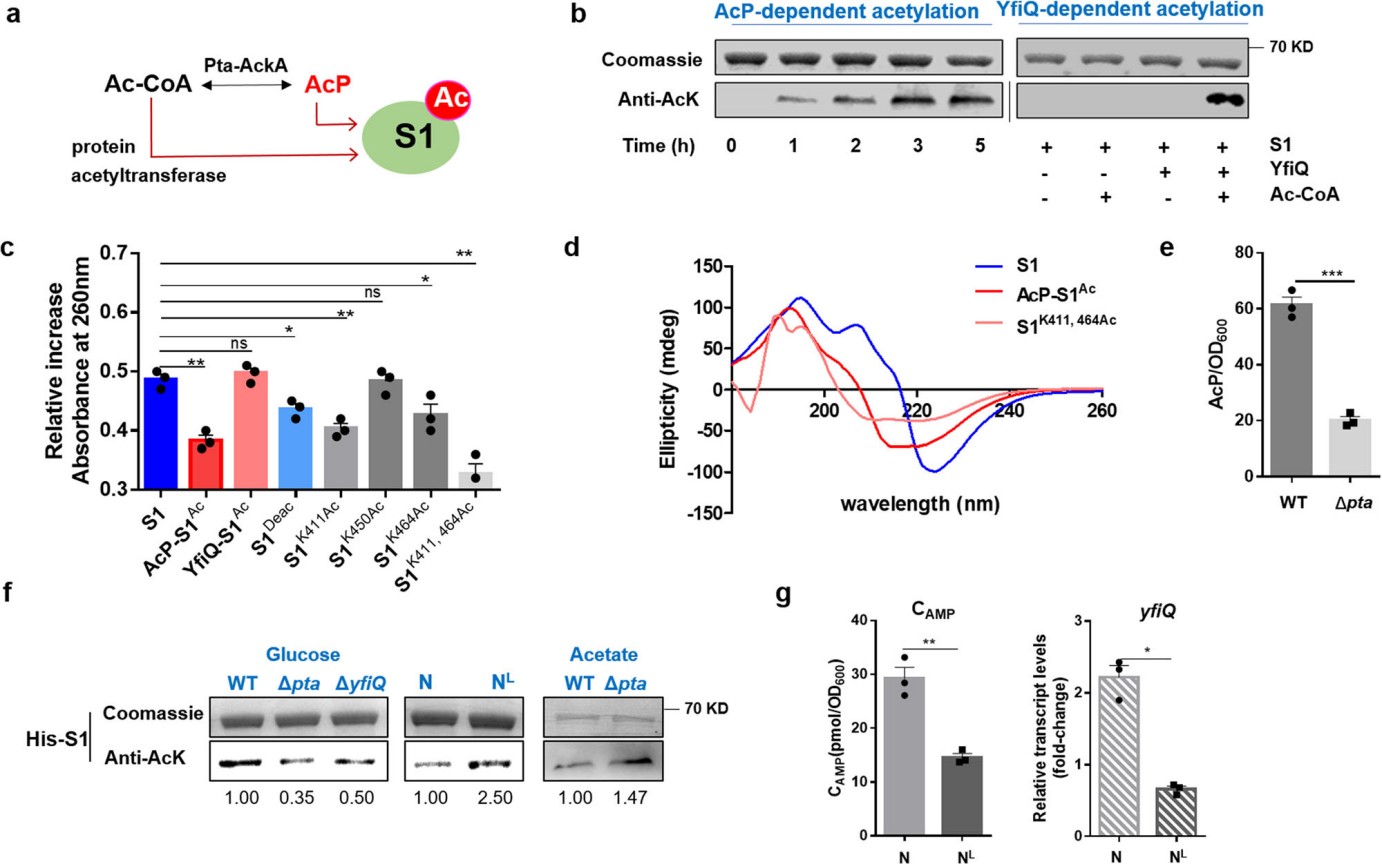
AcP-dependent acetylation of S1 regulated its function. **a** S1 acetylation via two mechanisms: enzymatic acetylation relies on a protein acetyltransferase (YfiQ) and chemical acetylation based on acetyl phosphate (AcP). **b** Purified His-S1 was acetylated by AcP or YfiQ in vitro. **c** Effect of S1 (WT-S1) and acetylated S1 (AcP-acetylated S1, AcP-S1^Ac^; YfiQ-acetylated S1, YfiQ-S1^Ac^; S1^Deac^, AcP-acetylated S1 was deacetylated by CobB; Site-specifically lysine acetylated proteins S1^K411Ac^, S1^K450Ac^, S1^K464Ac^ and S1^K411, 464Ac^) on the induction of hyperchromicity in poly(rC-U). *P < 0.05, **P < 0.01, ***P < 0.001. Three replicates were made for each sample. **d** Circular dichroism spectra of S1, AcP-S1^Ac^ and site-specifically lysine acetylated protein S1^K411, 464Ac^. **e** AcP concentrations of WT::S1 and Δ*pta*::S1 cultured in glucose. AcP concentration was normalized to OD_600_. *P < 0.05, **P < 0.01, ***P < 0.001. Three replicates were made for each sample. **f** Acetylation levels of overexpressed S1 in WT, *pta*-deleted mutant Δ*pta* and *yfiQ*-deleted mutant Δ*yfiQ* cultured in glucose or acetate; Acetylation levels of overexpressed S1 in *E. coli* cultured in N (standard nitrogen) or N^L^ (limited nitrogen). The band intensities were quantified by densitometry using Image J software. **g** Relative transcript levels of *yfiQ* and concentration of cAMP in *E. coli* cultured in N and N^L^ at 14 h. *P < 0.05, **P < 0.01, ***P < 0.001. Three replicates were made for each sample.

As an RNA molecular chaperone that plays a role in the recruitment of mRNAs to the ribosome, S1 has been found to be acetylated at some lysine sites in *E. coli*^18,29^. Recent studies have shown that protein acetylation can occur via two distinct mechanisms: enzymatic acetylation relies on a protein acetyltransferase (Pat) and chemical acetylation based on AcP, the high-energy intermediate of the phosphotransacetylase-acetate kinase pathway (Pta-Ack) (Fig. 1a)^30^. In this work, we found that *E. coli* protein S1 can be acetylated in vitro either catalytically by acetyltransferases YfiQ and Ac-CoA or nonenzymatically by AcP (Fig. 1b and Fig. S1). Both acetylated S1 proteins were partially deacetylated by deacetylase CobB (Fig. S2), which indicated reversible acetylation of S1. Protein S1 binds to the pyrimidine-rich region upstream of the mRNA SD sequence and recruits mRNA to ribosomes^31^. To examine the effect of S1 acetylation on its function, an RNA hyperchromicity assay was used to investigate the interaction of acetylated S1 with RNA^32^. As shown in Fig. 1c, it was clearly observed that the RNA hyperchromicity of S1 was reduced by AcP-induced acetylation and partially restored by CobB-catalyzed deacetylation; however, YfiQ-catalyzed acetylation had little effect. Circular dichroism assays also showed that AcP-induced acetylation significantly altered the secondary structure of S1 (Fig. 1d) (Table S1), revealing a decrease in parallel and random coil structures compared with native S1. These results suggested that AcP-induced acetylation of S1 might have a potential regulatory role in the recruitment of mRNAs by adjusting the interaction between S1 and mRNA.

We next explored the acetylation of S1 in vivo under different conditions. Lower acetylation levels of overexpressed S1 in *pta*-deleted mutant Δ*pta* with a low intracellular concentration of AcP when glucose was the sole carbon source (Fig. 1e, f)^33^ and *yfiQ*-deleted mutant Δ*yfiQ* were observed compared with the wild-type strain (Fig. 1f), which indicated that S1 can be acetylated in vivo by AcP or YfiQ. Since nitrogen starvation or acetate condition resulted in an accumulation of intracellular AcP and an increase in protein acetylation^33^, we assessed the acetylation level of S1 in *E. coli* cultured in N (standard nitrogen)/N^L^ (limited nitrogen) or acetate condition and found that nitrogen starvation and acetate condition induced the acetylation of S1 at stationary phase (Fig. 1f). Our previous works have shown that the intracellular AcP concentration in limited nitrogen was evidently higher than that in standard nitrogen^28^. Interestingly, the concentration of cAMP (which can activate the transcription of *yfiQ*) and the transcription level of *yfiQ* were lower under limited nitrogen (Fig. 1g). Taken together, these results showed that S1 acetylation induced by nitrogen starvation mainly depends on AcP-driven acetylation, not YfiQ catalysis.

The acetylated sites of S1 in vitro and in vivo were identified using mass spectrometry (Table S2), including K247, K411, K450, and K464 in the CTD domain. We found that only two lysine residues (411 and 464) are acetylated via the AcP pathway in Δ*yfiQ* strain. RNA hyperchromicity assay showed that YfiQ-catalyzed acetylation (K247 and K450) had no effect on the interaction between S1 and RNA (Fig. 1c). S1^K411Ac^, S1^K464Ac^ and S1^K411, 464Ac^ had obvious decrease in RNA hyperchromicity, while S1^K450Ac^ behaved similar as wild-type S1. The results was coincident with our previous work^28^ and it showed site-specifically lysine-acetylated protein S1 behaves as the lysine to glutamine mutations to mimic protein S1 acetylation. In addition, Circular dichroism assay also showed that S1^K411, 464Ac^ had a strong impact on the secondary structure of S1 (Fig. 1d and Table S1) and protein S1^K411Ac^ and S1^K464Ac^ were partially deacetylated by deacetylase CobB in the Western blot assay (Fig. S2c, d). These observations point to K411 and K464 as the key sites for AcP-driven acetylation in vivo and suggest that two lysine residues might be responsible for binding with mRNA.

### Acetylated S1 selectively recruits distinct subsets of mRNAs to ribosomes

**Fig. 2:**
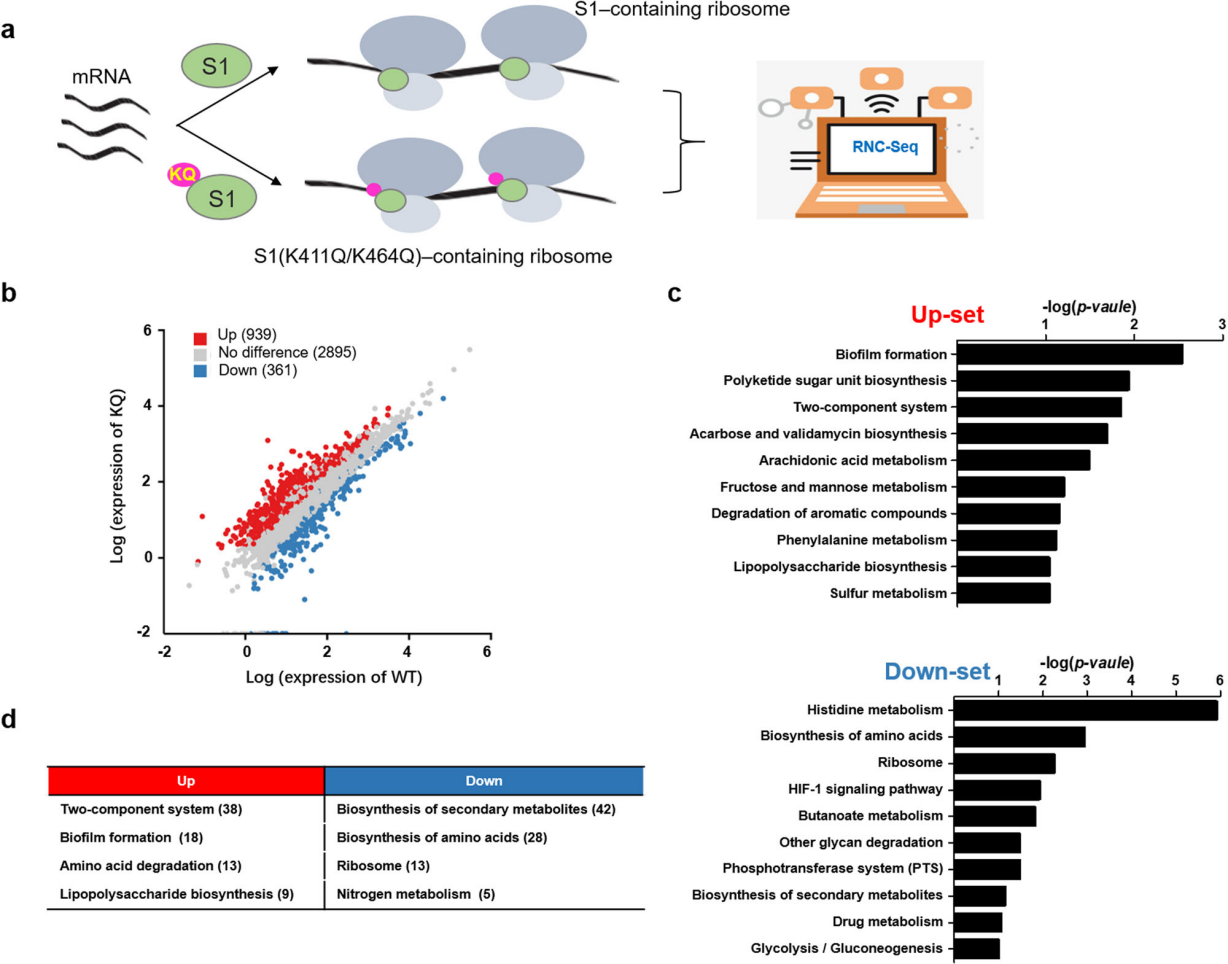
Acetylated S1 preferentially recruits different subpools of mRNAs to ribosomes. **a** Schematic of the ribosome-nascent-chain-complex sequence (RNC-Seq) of acetyl-mimetic S1 (K411Q/K464Q) or WT-S1-containing ribosomes. **b** Scatterplots for differentially expressed genes (up, red; down, blue.) in KQ/WT- RNC-Seq (*p* < 0.05). **c** KEGG pathway-based cluster analysis of KQ/WT-RNC-Seq upregulated genes and downregulated genes from **b**. **d** Significantly enriched KEGG pathways (*P* < 0.001) among Up-set or Down-set transcripts in KQ/WT-RNC-Seq. The key KEGG pathways are shown, and the number of associated genes in each KEGG pathway is shown in parentheses.

To investigate whether the acetylation of K411/K464 (S1K411Ac/S1K464Ac) plays a regulatory role in genome-wide translational control, an acetyl-mimetic (K411Q/K464Q) double mutant S1 was used to identify S1K411Ac/S1K464Ac-recruited mRNAs to ribosomes using the ribosome-nascent-chain-complex sequence (RNC-Seq) method (Fig. 2a). Two *E. coli* MG1655 strains engineered to overexpress S1 (WT) or S1 (K411Q/K464Q) were constructed (Fig. S3a). The overexpressed His-S1 or His-S1 (K411Q/K464Q) was obviously detected in the ribosome by western-blotting assay (Fig. S3b). Translating mRNA fragments in S1 (WT)- or S1 (K411Q/K464Q)-containing ribosomes (Fig. S3b) were isolated and sequenced after removing ribosomes to quantify and compare the mRNAs recruited by S1 and S1 (K411Q/K464Q). Three biological replicates were generated for each RNC-Seq library (KQ, WT), which showed high consistency with each other (Fig. S3c).

RNC-Seq revealed a significant shift and bias for translating mRNAs engaged in ribosomes containing S1 and S1 (K411Q/K464Q), indicating that acetyl-mimetic mutations of K411 and K464 had an important effect on global translation (Fig. 2b). Two subsets of transcripts were observed: one subset (Up-set, 939 transcripts) exhibited upregulation; the other subset (Down-set, 361 transcripts) exhibited downregulation in ribosomes with S1 (K411Q/K464Q) compared with S1 (Fig. 2b). These transcripts were statistically significant (*P* < 0.001) and met the threshold of fold change (FC) (|log_2_FC|>1).

The Up-set or Down-set of transcripts were enriched in different KEGG pathways. Interestingly, we observed that the enriched pathways of the two sets tended to exhibit opposite functions in cellular metabolism (Fig. 2c, d). It is particularly noteworthy that many pathways that emerged from the KEGG analysis of the two sets were involved in the nutrient-stress response, amino acid metabolism, and nitrogen metabolism. For example, the enriched pathways of Up-set transcripts include biofilm formation for nutrient-stress response, two-component system for the transport and assimilation of nutrients, and amino acid degradation for supply of nitrogen source. In contrast to Up-set transcripts, Down-set transcripts were enriched in nitrogen-consuming processes, such as biosynthesis of amino acids and RPs. These findings suggested that nitrogen starvation-induced acetylation of K411/K464 might be responsible for preferential recruitment of specific stress-responsive mRNAs to ribosomes as translation selectors.

### Acetylated S1-mediated translational reprogramming for adaptation to nitrogen starvation

**Fig. 3:**
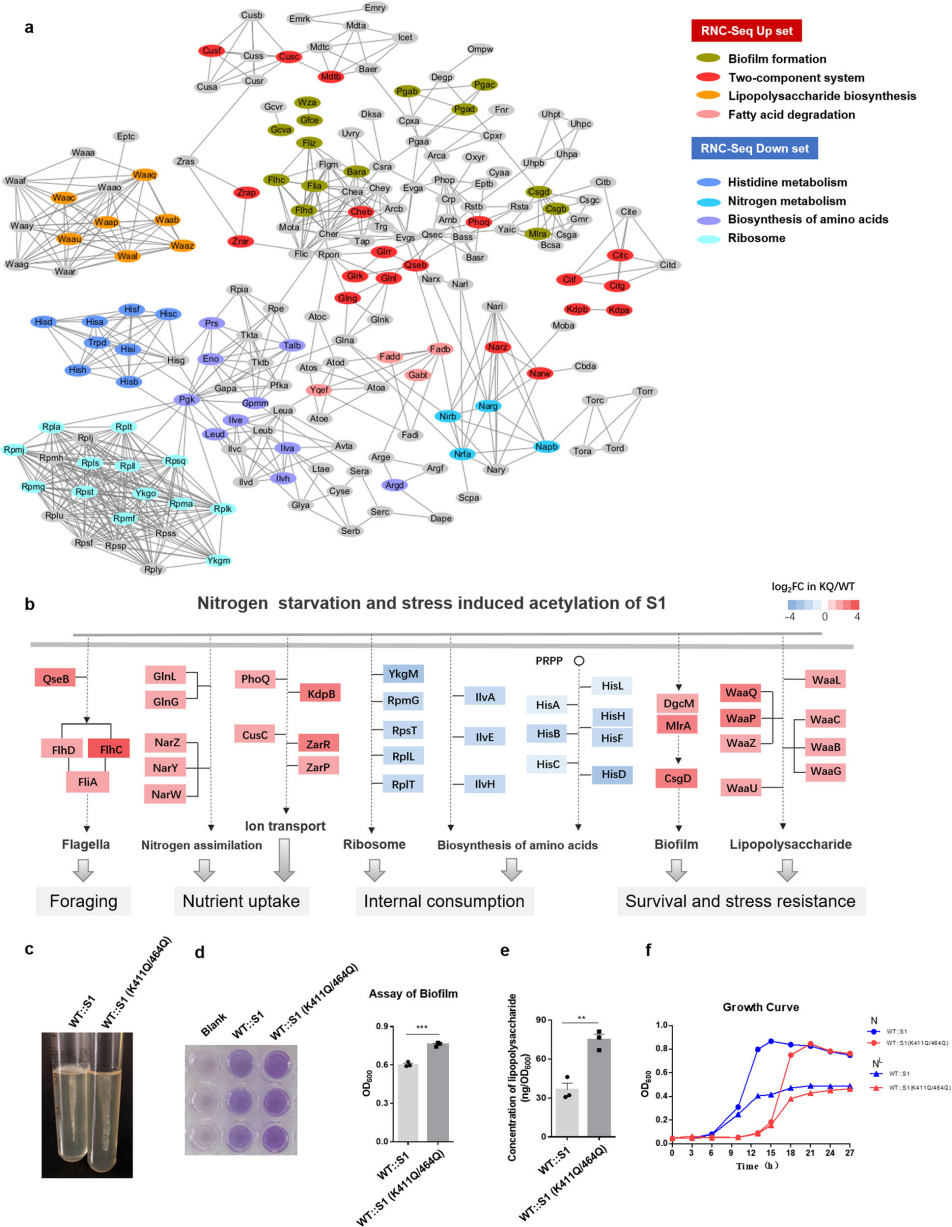
Coordinated translational regulation of genes with related biological functions by S1 (K411Q/K464Q)-containing ribosomes. **a** Network-based cluster analysis of KQ/WT-RNC-Seq up- or downregulated genes and their associated functional classes. **b** Schematic diagram of signal transmission based on pathways enriched in RNC-Seq up- or downregulated genes. **c** Motility assay of WT::S1 and WT::S1 (K411Q/K464Q) on semisolid medium. **d** Biofilm assay of WT::S1 and WT::S1 (K411Q/K464Q) in a 96-well microplate. *P < 0.05, **P < 0.01, ***P < 0.001. Three replicates were made for each sample. **e** Lipopolysaccharide measurement of WT::S1 and WT::S1 (K411Q/K464Q). *P < 0.05, **P < 0.01, ***P < 0.001. Three replicates were made for each sample. **f** Growth curves of WT::S1 and WT::S1 (K411Q/K464Q) cultured in nitrogen-rich (N) or nitrogen-limited (N^L^) conditions. Three replicates were made for each sample.

An acetylated S1-mediated translational shift may be critical to tailor coordinated translation reprogramming to adapt to nitrogen starvation. Network-based cluster analysis of Up-set and Down-set genes and their associated functional classes was performed. Interestingly, S1 (K411Q/K464Q)-regulated genes showed strong correlations and exhibited an obvious enrichment in nutrient metabolism and stress-response pathways (Fig. 3a). Indeed, Up-set included a general bacterial nutrient-stress response involved in FlhD-FlhC/FliA-driven flagellar activity and growth for the foraging strategy at low nutrient levels, as well as DgcM/MlrA-CsgD-dependent biofilm formation and multicellularity for the survival strategy under extreme nutrient limitation (Fig. 3b)^1^. As shown in Fig. 3c, d, the strains containing S1 (K411Q/K464Q) showed stronger motility activity on semisolid agar plates and produced more biofilms by static culture in 96-microplate plates. In bacteria, the complex and sophisticated two-component system (TCS) senses, conducts, and responds to environmental stress. As expected, we found that many transcripts functioning in the two-component system were enriched in S1 (K411Q/K464Q)-mediated Up-set (Fig. 3b). For example, RstB/A responding to stress and QseB/C regulating flagellar activity and biofilm formation were upregulated by 5.6/2.3-fold and 4.9/1.4-fold, respectively, in the *E. coli* strain with S1 (K411Q/K464Q) (Table S3). GlnL/G and NarW/Y/Z, which are involved in nitrogen assimilation, and GlrK/R, which is involved in amino sugar metabolism, were preferentially recruited and translated by acetyl-mimetic S1 (Table S3). Many key TCSs and related proteins involved in ion transport revealed upregulated expression, including PhoQ/PhoP transporting Mg^2+^ ions, KdpA/KdpB transporting K^+^ ions, CusF/CusC for efflux of Cu^2+^ ions, and ZraR/ZraP for efflux of Zn^2+^ and Pb^2+^ ions (Table S3). Under nutrient-depleted conditions, bacteria enter the stationary phase and produce extracellular matrix components and lipopolysaccharides, which are related to biofilms and cell walls, thereby promoting stress resistance. Introduction of acetyl-mimetic S1 resulted in much increased expression of lipopolysaccharide biosynthesis (Table S3) and an increase in the amount of lipopolysaccharide by ~50% (Fig. 3e) compared to wild-type S1. Furthermore, deletion of the *pta* gene induced acetylation of S1, enhancing biofilm formation and lipopolysaccharide biosynthesis (Fig. S3d, e). On the other hand, Down-set genes function in some cellular pathways involved in the biosynthesis of amino acids and ribosomes, biosynthesis of secondary metabolites, and carbon metabolism. Remarkable examples are histidine biosynthesis and RPs. Many genes in the operon of histidine biosynthesis were downregulated in the strain with S1 (K411Q/K464Q) (Table S3). Thirteen transcripts encoding RPs exhibited lowered translation activities by acetyl-mimetic S1 (Figs. 2d and 3a), thereby inhibiting global translation. The strains with acetyl-mimetic S1 had a delayed effect on growth (Fig. 3f). Altogether, acetylated S1-mediated translational reprogramming revealed an overall tendency toward saving and reallocating cellular resources for survival. In summary, these results solidified acetylation of S1 as a translational selector (recruiter, selectively recruits different subpools of mRNAs) for translational reprogramming to adapt to nitrogen starvation.

### S1-binding sequence (SBS) plays a crucial role in acetylated S1-mediated translational regulation

**Fig. 4:**
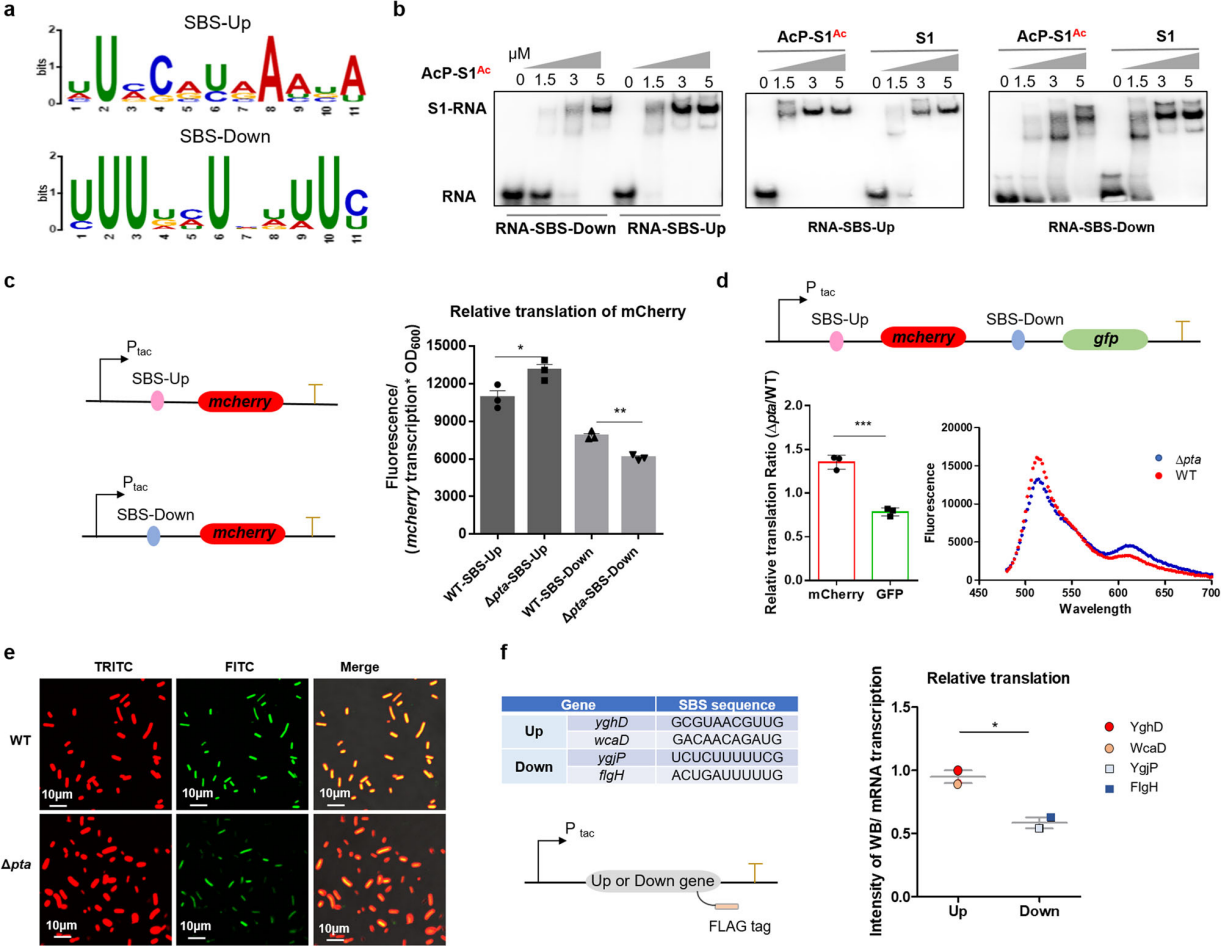
The S1-binding sequence (SBS) plays a crucial role in acetylated S1-mediated translational regulation. **a** Analysis of conserved sequences of the top 50 up- or downregulated genes regulated by acetyl-mimetic S1. **b** Mobility-shift assay gels for the binding of S1 (WT) or AcP-S1^Ac^ with RNA containing SBS-Down (UUUUCUCUUUC) and RNA containing SBS-Up (UUCCAUAAAUA). **c** Relative translation of mCherry mRNA with SBS-Up or with SBS-Down in WT or Δ*pta* strain. The relative translation is equal to the relative fluorescence unit (RFU)/the relative transcription level, RFU is equal to Fluorescence/OD_600_. *P < 0.05, **P < 0.01, ***P < 0.001. Three replicates were made for each sample. **d** Relative translation of SBS-Up-guiding mCherry and SBS-Down-guiding GFP in WT or Δ*pta* strain when cultured in acetate. *P < 0.05, **P < 0.01, ***P < 0.001. Three replicates were made for each sample. **e** Intracellular mCherry and GFP in WT and Δ*pta* strains when cultured in acetate using confocal laser scanning microscopy. Scale bar: 10 μm. **f** Relative translation levels of two target transcripts (*yghD* and *wcaD*) in Up-set and two target transcripts (*ygjP* and *flgH*) in Down-set in the Δ*pta* strain when cultured in acetate. *P < 0.05, **P < 0.01, ***P < 0.001. Three replicates were made for each sample.

We next investigated whether acetylated S1-mediated translational regulation could be influenced by specific motifs embedded in the sequences of mRNAs. RP S1 interacts with approximately 11 nucleotides (nt) of mRNA immediately upstream of the Shine-Dalgarno (SD) sequence^34^. To explore the effect of 11-bp sequences (designated S1-binding site, SBS) on translational regulation, we compared the characteristics of SBS sequences in the 50 transcripts in Up-set or Down-set using MEME (Multiple EM for Motif Elicitation). Enriched A/U-containing sequences (SBS-Up motif: UUCCAUAAAUA, SBS-Down motif: UUUUCUCUUUC) were observed (Fig. 4a). In particular, a highly conserved U-rich motif (UUUxxUxxUUx) was identified upstream of the SD sequence of Down-set transcripts. We speculated that S1 or AcP-induced acetylated S1 might reveal different binding affinities with mRNAs with different SBSs, thereby selectively promoting or inhibiting the translation of these mRNAs. To gain direct evidence for the influence of S1 acetylation on S1-mRNA interactions, EMSA was performed to compare the binding affinity of S1 or AcP-acetylated S1 (AcP-S1^Ac^) with SBS-Up-containing or SBS-Down-containing RNA. As shown in Fig. 4b, the results demonstrated that natural S1 and acetylated S1 had different binding affinities to RNA-SBS-Up or RNA-SBS-Down: natural S1 preferentially bound RNA-SBS-Down compared to RNA-SBS-Up; acetylation of S1 led to an increase in binding affinity with RNA-SBS-Up and a decrease in binding affinity with RNA-SBS-Down.

Protein S1 was demonstrated to be hyperacetylated under nitrogen starvation or acetate as the carbon source (Fig. 1f). To avoid the negative effects caused by nitrogen starvation on the bacterial growth, acetate was used as the carbon source to test the effect of acetylated S1 on the characteristic SBS guiding translation assay. To gain more insight into the effect of S1 acetylation on the translation of mRNAs with SBS-Up motifs or SBS-Down motifs in vivo, we constructed plasmids in which two SBS motifs were incorporated into dual-fluorescent protein reporters in the WT strain and Δ*pta* strain, where the acetylation level of S1 was higher on acetate as a carbon source. The expression of mCherry was guided at the same P_tac_ (a modified constitutive promoter) and RBS in pUC19 (Fig. 4c). The relative transcription level and fluorescence of mCherry were measured to normalize the relative translation (protein/mRNA transcripts) (Fig. S4a). Importantly, the relative translation of mCherry mRNA with SBS-Up was higher in Δ*pta* than in WT (Fig. 4c). In contrast, the relative translation of mCherry mRNA with SBS-Down was lower in Δ*pta* than in WT. These results indicate that S1, with a higher acetylation level in Δ*pta*, has a higher affinity with SBS-Up than SBS-Down, which is consistent with the EMSA in vitro (Fig. 4b). To further eliminate the interference of strains, we coupled SBS-Up-guiding mCherry and SBS-Down-guiding green fluorescent protein (GFP) in pUC19 (Fig. 4d), which overcame slight differences in transcriptional levels. The relative translation ratio (Δ*pta*/WT) of mCherry was higher than that of GFP (Fig. 4d, e). As AcP accumulation caused global hyperacetylaion more than one S1, to confirm whether the effects observed are due to S1 acetylation, we used S1 (K411Q/K464Q) and S1 overexpressed strains and found that the *rpsA* transcription levels after IPTG induction were comparable and significantly higher than the transcription of background S1 under identical culture conditions (Fig. S5a). In addition, the dual-fluorescent assay and the relative translation of SBS-Up/Down guiding mCherry in the S1 (K411Q/K464Q) and S1 overexpressed strains also revealed highly consistent trend with Fig. 4 (Fig. S5b, c). These results supported that the effects in Fig. 4 above had a high correlation with S1 acetylation, in other words, S1 acetylation played a major role in this regulation.

Next, we chose two target transcripts (*yghD* and *wcaD*) in Up-set and two target transcripts (*ygjP* and *flgH*) in Down-set. These four target genes with a Flag tag were inserted into pUC19 plasmids and expressed in the Δ*pta* strain cultured in acetate (Fig. 4f). The transcriptional levels (RT-qPCR) and protein amounts (Western-bloting assay) of the target genes were determined (Fig. S4b, c). Quantification of relative translation showed a significant difference in the protein outputs of *ygjP* and *flgH* compared to *yghD* and *wcaD* (Fig. 4f). Previous works have shown that an A/U-rich sequence upstream of the RBS (ribosome-binding site) facilitates translation^35,36^. Our findings strongly point to a direct role for SBS in acetylated S1-mediated translational regulation depending upon the differential binding affinities of S1 and acetylated S1 to SBS of distinct mRNAs.

### The specific SBS can be employed to dynamically fine-tune gene expression

**Fig. 5:**
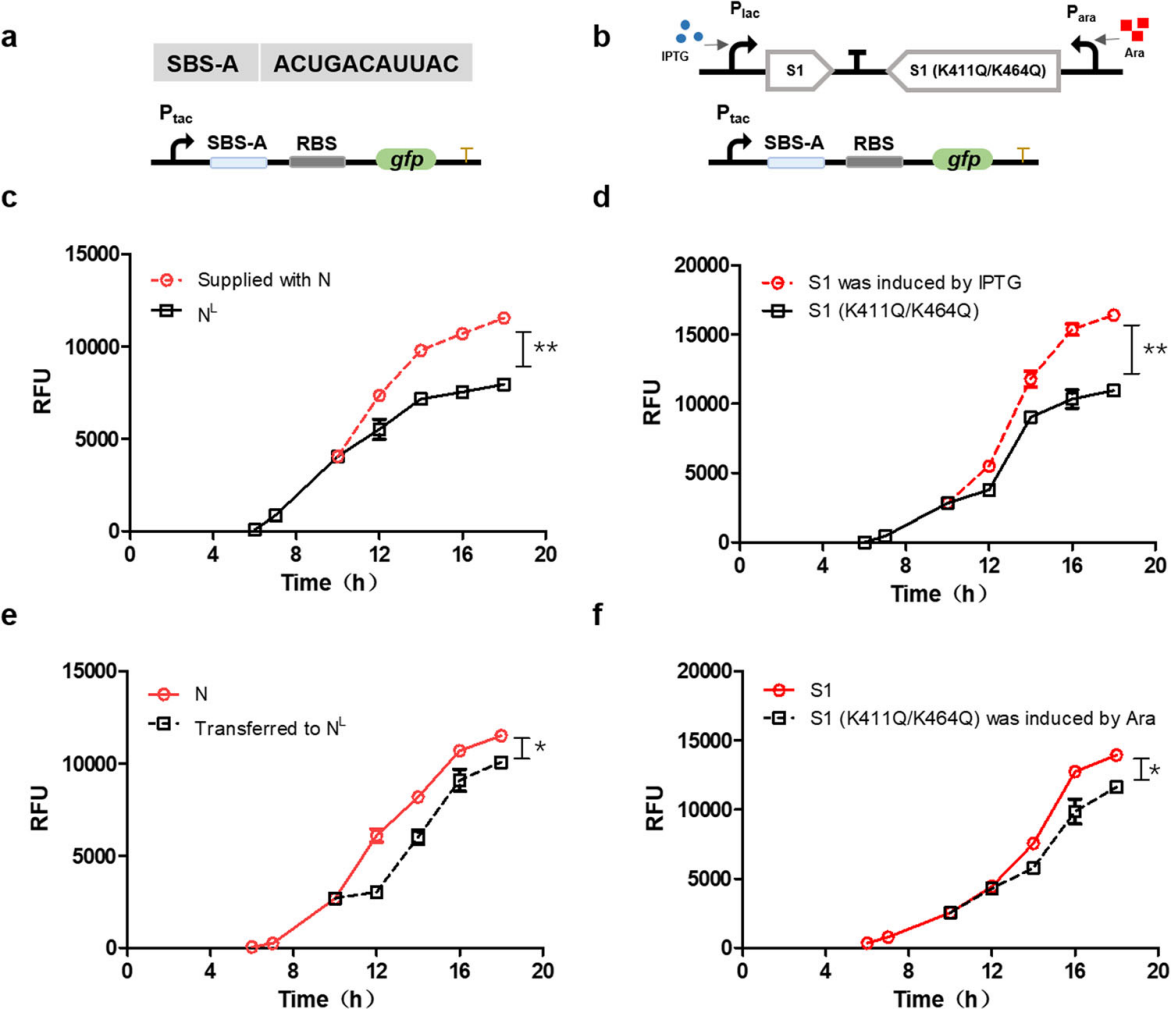
The specific SBS can be employed to dynamically fine-tune gene expression. **a** Sequence of SBS-A and schematic of the SBS-A testing system (pKD236-P_tac_-SBS-A-GFP) in *E. coli* BL21. **b** *E. coli* BL21 with pET-28a-S1 (induced by IPTG) and acetyl-mimetic S1 (induced by arabinose). **c** Expression of GFP (relative fluorescence unit) in *E. coli* BL21 cultured in N^L^ or supplemented with nitrogen (N) at 10 h. The black line represents the RFU of GFP in *E. coli* BL21 cultured in N^L^, the red line represents the RFU of GFP in *E. coli* BL21 after addition of nitrogen in N^L^ at 10 h. *P < 0.05, **P < 0.01, ***P < 0.001. Three replicates were made for each sample. **d** Expression of GFP in *E. coli* BL21 cultured in N or transferred to limited nitrogen (N^L^) at 10 h. The red line represents the RFU of GFP in *E. coli* BL21 cultured in N, the black line represents the RFU of GFP in *E. coli* BL21 after transferring to N^L^ at 10 h. *P < 0.05, **P < 0.01, ***P < 0.001. Three replicates were made for each sample. **e** Expression of GFP in *E. coli* BL21 with pET-28a-S1-S1 (K411Q/K464Q) when cultured in LB medium. Arabinose was added to induce S1 (K411Q/K464Q) at 0 h, and IPTG was added to induce S1 at 10 h. The black line represents the RFU of GFP in *E. coli* BL21 with arabinose, the red line represents the RFU of GFP in *E. coli* BL21 after adding IPTG at 10 h. *P < 0.05, **P < 0.01, ***P < 0.001. Three replicates were made for each sample. **f** IPTG was added to induce S1 at 0 h, and Ara was added to induce S1 (K411Q/K464Q) at 10 h. The red line represents the RFU of GFP in *E. coli* BL21 with IPTG, the black line represents the RFU of GFP in *E. coli* BL21 after adding arabinose at 10 h. *P < 0.05, **P < 0.01, ***P < 0.001. Three replicates were made for each sample.

The ribosome-binding site (RBS), one of the key elements that regulates the translation level, has widely been investigated. In this work, we found that SBS is also a key element to fine-tune the translational level of proteins via acetylation of S1 in response to environmental stress. To explore and identify SBSs with various strengths to S1 or acetylated S1, an SBS library was constructed into pKD236 containing a modified constitutive promoter P_tac_ and RBS (AAGGAG) and tested in *E. coli* BL21 possessing pProEX-S1 or pProEX-S1 (K411Q/K464Q) (Fig. S6a). A total of 172 clones in the pProEX-S1 library and 177 clones in the pProEX-S1 (K411Q/K464Q) library were selected with different fluorescence intensities (Fig. S6) (The more details were shown in Supplementary Data 1). To investigate the effect of the SBS element on gene expression, a random SBS sequence (SBS-A, ACUGACAUUAC) in the SBS library was used to dynamically fine-tune the expression of GFP (Fig. 5a, b). The addition of nitrogen resulted in an increase in GFP fluorescence intensity (relative fluorescence unit) of *E. coli* BL21 with pKD236-P_tac_-SBS-A-GFP cultured in nitrogen-limited conditions (Fig. 5c). The depletion of nitrogen led to a decrease in the GFP fluorescence intensity of *E. coli* BL21 with pKD236-P_tac_-SBS-A-GFP cultured in nitrogen-rich conditions (Fig. 5d). Furthermore, to eliminate the growth difference caused by supplementary nutrition, the effect of SBS was examined in the S1 (induced by IPTG) and acetyl-mimetic S1 (K411Q/K464Q) (induced by arabinose, Ara) systems (Fig. 5b). We found that S1 induced by IPTG significantly improved the expression of GFP (Fig. 5e), whereas acetyl-mimetic S1 induced by arabinose repressed the expression of GFP (Fig. 5f). The results indicated that the various combinations of SBS and S1 can be harnessed to fine-tune gene expression.

## Discussion

**Fig. 6:**
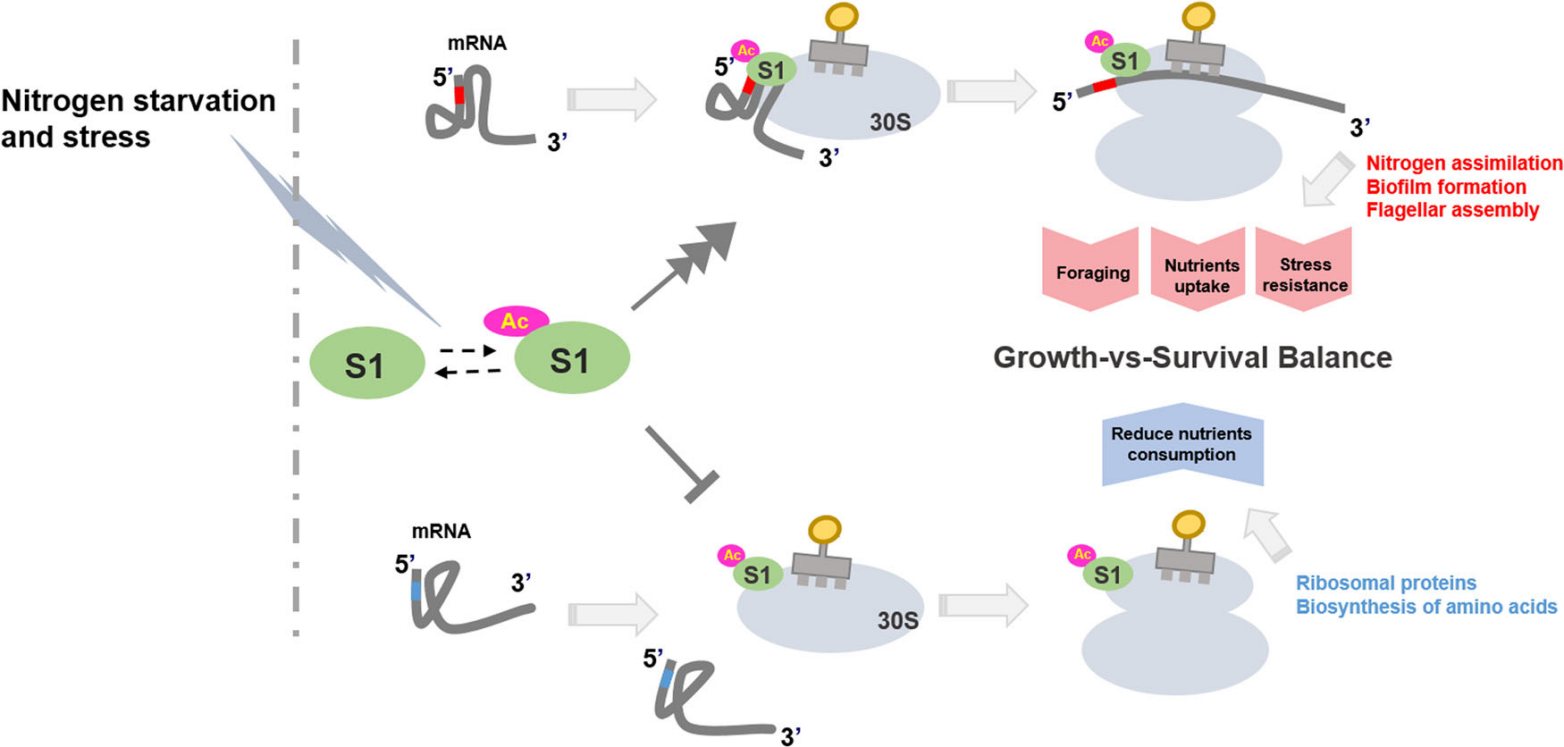
Coordinated regulation of S1 acetylation-driven translation reprogramming for cell survival during nitrogen starvation. The dotted arrows represent the reversible acetylation of S1, solid arrows in dark gray represent the upregulated recruitment of mRNA and the perpendicular lines represent the downregulated recruitment.

We have previously demonstrated that the acetylation of translation machinery inhibits global translation in *E. coli*^28^. Herein, we uncovered a molecular mechanism leading to translational reprogramming upon acetylation of RP S1 as a translation selector under stressful conditions. We show that AcP-induced S1K411/464Ac, as nitrogen starvation stress S1, selectively enhances or inhibits translation of subsets of mRNAs for adapting to environmental stress. The translational selectivity depends on the binding affinities of S1K411/464Ac with SBS sequences in the 5′-UTR of mRNAs. Nitrogen starvation-induced the accumulation of intracellular AcP, which drove the acetylation of S1 and resulted in translational reprogramming. We found that acetylated S1-mediated translation reprogramming does not cause a complete change but a crucial modulation of the translation program, thereby adjusting cellular metabolism to the nutrient status (nitrogen starvation) of the cell. The transcripts upregulated by S1K411/464Ac included general nutrient-stress-responsive pathways, such as biofilm formation for stress resistance, flagella assembly for food foraging, and a two-component system for the transport and assimilation of nutrients, as well as pathways involved in amino acid degradation. The transcripts downregulated by S1K411/464Ac are enriched in nitrogen-consuming processes, such as biosynthesis of amino acids and RPs, thereby allowing bacteria to enter the stationary phase. These findings suggested that nitrogen starvation-induced acetylation of S1 leads to translational reprogramming specifically tailoring to nitrogen starvation stress, which is designated the “nitrogen starvation-responsive program” (Fig. 6).

Bacteria have evolved diverse regulatory mechanisms responding to the availability of nutrients and adapting to nutrient deficiencies at both the transcriptional and translational levels. Protein-synthesizing ribosomes can effectively and economically integrate environmental signals into the regulatory network of protein synthesis and therefore lie at the heart of cellular adaptation in response to nutrient stress. We found that many key regulators and pathways in this “nitrogen starvation-responsive program” were observed in the highly integrated regulatory network controlling the growth-versus-survival balance in *E. coli*. Acetylation-regulated S1 mediates the translational response, allowing cells to reallocate cellular resources for growth and proliferation or for survival (biofilm and multicellularity) depending on nutrient availability (Fig. 6). Together, the identification of S1K411/464Ac and its ability to preferentially translate stress-responsive subsets of mRNAs reveals an important additional layer of the integrated regulatory network regulating the cellular response to stress.

Hundreds of chemical modifications have been identified on the ribosome to generate numerous heterogeneous and functionally specialized ribosomes^15^, allowing the cells to undergo near-immediate translation reprogramming for survival during sudden changes in their environment. Protein synthesis has a great demand for cellular resources^19^. An effective and economical strategy of translational regulation should be the selective recruitment and initiation of stress-responsive mRNAs using specific modified translation initiation factors. The molecular mechanisms based on the chemical modification of eIFs have been revealed to lead to either global or transcript-specific control of translation initiation^21,23^. Here, we discovered that bacteria possess a hitherto uncharacterized stress adaptation mechanism based on reversible acetylation of S1 to shape the proteome during stress to guarantee bacterial survival by selectively recruiting specific mRNAs.

In bacteria, RBS sequences with various strengths have been screened, identified, and standardized (http://parts.igem.org/Ribosome_Binding_Sites) and have been harnessed as effective regulatory elements to control gene expression for metabolic engineering and synthetic biology. The strength of RBS on an mRNA influences its rate of translation initiation. SBS exerts an effect on which mRNAs are preferentially translated. Therefore, SBS should be taken into consideration as an important regulatory element for fine-tuning protein translation. It is desired that many standardized SBSs with different preferences and strengths be identified and designed. In this study, a series of SBS sequences to S1 and acetylated S1 were investigated and collected as the SBS Database (Supplementary Data 1), providing a means for dynamically selective synthesis of specific proteins.

Altogether, this work provides a paradigm for translational stress response in bacteria based on functionally specialized S1: the heterogeneity of the translational machinery caused by AcP-induced chemical modification results in a systems-level remodeling of specific networks of mRNAs, as the “nitrogen starvation-responsive program”. These findings highlight the potential diversity in RPs with various dynamic PTMs to expand the functional role of the heterogeneous ribosome and to define different layers of control for protein synthesis.

## Methods

### Bacterial strains, growth conditions, and reagents

*E. coli* MG1655 wild-type and mutant strains were used in this study and listed in Table S4. The selected *E. coli* strains were grown in lysogeny broth (LB) medium or minimal-salts medium supplemented with 0.2% (w/v) glucose or 0.27% (w/v) acetate, 6.25 mM (NH_4_)_2_SO_4_ (standard nitrogen concentration, N), or 1.56 mM (NH_4_)_2_SO_4_ (limited nitrogen concentration, N^L^)^28^ at 37 °C on a rotary shaker at 220 rpm. Growth was monitored by measuring the optical density at 600 nm (OD_600_). Acetyl-lysine antibody (PTM-102) was purchased from PTM BioLab (HangZhou Jingjie).

### Site-directed mutagenesis of protein S1

The acetylated-sites mutant of protein S1 (S1 (K411Q/K464Q) or S1 (WT) was introduced into the pProEX-HTb. The primers used in this study are listed in Table S5. Recombinant plasmids were transferred to *E. coli* MG1655 (WT), Δ*pta,* and Δ*yfiQ*, ampicillin-resistant transformants were selected on LB agar medium with 100 µg/mL ampicillin.

### Overproduction and purification of protein S1 in vitro

*E. coli* was cultured overnight in 5 mL LB and then transferred to 100 mL LB medium supplemented with 1‰ kanamycin (50 mg/mL) or 1‰ ampicillin (100 mg/mL). The cells were grown at 37 °C to OD_600_ ~0.5 and 1 mM IPTG was added to induced protein expression at 20 °C for 16–20 h. After induced, cells were harvested by centrifugation, washed twice with 20 mM ice-cold PBS buffer (pH 8.0) and broken by sonication in PBS. Protein S1 was purified by Ni-NTA Superflow columns (Qiangen, Valencia, CA), S1 concentration was monitored by the BCA method using buffer PBS as the control and the standard curve was determined by BSA. S1 purified from BL21 was used to AcP and YfiQ acetyltransferases driven acetylation in vitro. S1 purified from WT::S1, Δ*yfiQ*::S1 and Δ*pta*::S1 were directly used to identify acetylation level by western blot (WB). The acetylated sites of S1 were identified by mass spectrometry^37^.

The site-specifically lysine-acetylated S1 proteins (S1^K411Ac^, S1^K450Ac^, S1^K464Ac^, and S1^K411, 464Ac^) were prepared using genetic code expansion according to a previous report^38^. In brief, *Escherichia coli* BL21 cells were transformed with plasmid pTECH-MbAcK3RS(IPYE) (encoding MbAcK3RS(IPYE) and pylT) and pET-28a (+) (carrying the S1 ORF with an amber codon (TAG) at the desired sites). The cells were first grown overnight in 5 ml LB medium supplemented with 50 mg/mL kanamycin and 50 mg/mL chloramphenicol (LB-KC) at 37 °C. In all, 300 ml LB-KC was inoculated with 2 ml overnight culture and was incubated at 37 °C. At OD_600_ of 0.5~0.6, the culture was supplemented with 10 mM nicotinamide (NAM) and 5 mM acetyl-lysine. After 30 min, 0.5 mM IPTG was added to induce protein expression at 20 °C for 14–16 h. Cells were harvested by centrifugation at 4 °C, and were washed with ice-cold PBS containing 20 mM NAM. The proteins were purified as above.

pTECH-MbAcK3RS(IPYE) was a gift from David Liu (Addgene plasmid # 104070; http://n2t.net/addgene:104070; RRID:Addgene_104070).

### In vitro acetylation and deacetylation of S1

For AcP-driven acetylation, 10 mM AcP was used to mimic the physiological condition to acetylate S1 at 37 °C for 3 h. To determine whether S1 was a substrate for YfiQ, 20 µg purified S1 was incubated with 0.5 µM purified YfiQ and 20 µM Ac-CoA (200 µL total volume) containing 0.05 M HEPES buffer (pH 7.5) at 37 °C for 1.5 h. The samples were divided into two portions: one portion was resolved and analyzed by western blot^37^, and the other was used for deacetylation assay. Deacetylation assay was performed in the presence of 1 mM NAD^+^ and 0.2 µM deacetylase CobB. After the reaction, acetylated sites were analyzed by mass spectrometry.

### RNA hyperchromicity assay

RNA hyperchromicity of S1 or acetylated S1 was described in detail in our work^28^. RNA hyperchromicity refers to the increase of absorbance at 260 nm when RNA molecules are denatured or broken. As an RNA-affinity protein, S1 can destroy the secondary structure of RNA and increase the absorbance of RNA at 260 nm. For RNA hyperchromicity, 10 μl protein S1 (2.0 μM) was added into a reaction mixture (40 μl total volume) containing 5 mM Tris-HCl (pH 7.4), 10 mM NaCl, and 240 μg/μl Poly(rC-U) at room temperature. The absorbance was measured at 260 nm.

### Mass spectrometry peptide fingerprinting

Briefly, protein S1 was cut off from the Coomassie-stained gel and washed with 50% ethanol, the sample was cut into 1 mm^3^ pieces and reduced with 10 mM dithiothreitol at 56 °C for 1 h, then 55 mM acrylamide was added and the sample was incubated for 45 min in darkness at room temperature. The protein was digested with 10 ng/μl trypsin (Promega) in 50 mM NH_4_HCO_3_ for 16 h at 37 °C. Tryptic digests were extracted with 50% ACN, 5% TFA (v/v) solution and 75% ACN, 0.1% TFA (v/v) solution, respectively. The peptides were desalted with a desalting column and dissolved in 0.1% TFA (v/v). The peptides were identified by mass spectrometry strictly according to the procedure described by You et al^37^. MS/MS spectra were searched using MASCOT engine (Matrix Science, London, UK; version 2.2) against Uniprot *E.coli* K-12 MG1655 database. Protein identification options: Peptide mass tolerance=10 ppm, LC-MS/MS tolerance = 0.02 Da, Enzyme=Trypsin, Missed cleavage = 2, Fixed modification: Carbamidomethyl (C), Variable modification: Oxidation (M), Acetylation (K, N-terminal). The protein identification data false discovery rate (FDR) of ≤1%. The MS data was shown in Fig. S7.

### Circular dichroism spectrometry assay

WT-S1, AcP-acetylated S1, and site-specifically lysine-acetylated protein S1^K411, 464Ac^ were evaluated using circular dichroism spectrometry (Applied Photophysics, Leatherhead, United Kingdom) in the far-UV region (180–260 nm) at room temperature using a 10-mm cuvette. The proteins (0.2 mg/mL) were dissolved in a modified PBS buffer (pH 7.4) contained 1.4 M KF, 100 mM K_2_HPO_4_, 18 mM KH_2_PO_4_. The circular dichroism spectrum scan of every sample was performed in triplicate. The spectra were analyzed for secondary structure content using CDNN CD spectra deconvolution software.

### Measurement of cAMP, RNA preparation, and RT-PCR

WT::S1 was cultured in 100 mL minimal-salts medium supplemented with 0.2% (w/v) glucose and 6.25 mM (NH_4_)_2_SO_4_ (standard nitrogen concentration, N), or 1.56 mM (NH_4_)_2_SO_4_ (limited nitrogen concentration, N^L^) for 14 h. 20 mL stationary phase cultures were harvested by centrifugation for cAMP activity assay according to cAMP ELISA Kit (D770001, Sangon). For RNA preparation, 10 mL stationary phase cultures were centrifuged at 4 °C and cell pellets were washed with RNase-free water. Total RNA was prepared using RNAprep pure Cell/Bacteria Kit (Tiangen Biotech Co., Ltd., Beijing, China, DP430) and was reverse transcribed as mentioned in our previous work^37^. PCR reactions were performed with primers listed in Table S5. For real-time RT-PCR, TransStart® Green qPCR SuperMix (Transgen) was used and about 100 ng cDNA was added in 20 µL volume of PCR reaction. The PCR was conducted using CFX96 real-time system (Bio-Rad, USA), and the PCR conditions were 95 °C for 5 min, then 40 cycles of 95 °C for 5 s, 60 °C for 30 s.

### RNC-Seq assay

RNC-Seq details are described below^39,40^. WT::S1 and WT::S1 (K411Q/K464Q) were grown in 100 mL liquid LB at 37 °C on a rotary shaker at 220 rpm to OD_600_~0.4 and 1 mM IPTG was used to induce the expression of S1, after 2.5 h, chloramphenicol (100 µg/mL) was added to stop mRNA translation before collecting cells. The cells were quickly collected by centrifugation at 4 °C and washed twice with 5 mL pre-cooled buffer A (10 mM MgCl_2_, 100 mM NH_4_Cl, 20 mM Tris pH 8.0, 1 mM chloramphenicol). The cell pellet was stored at −80 °C or directly used for ribosome extraction. For ribosome extraction, cells were suspended and lysed in 3.75 mL pro-cooled buffer B (10 mM MgCl_2_, 100 mM NH_4_Cl, 20 mM Tris pH 8.0, 0.1% NP-40, 0.4% triton-100, 100 U/mL RNase-free DNase I (Takara Bio), 5 U/µL RNase inhibitor (Sangon) and 1 mM chloramphenicol). After incubated on ice for 5 minutes, the suspended cells were dripped into a mortar with liquid nitrogen and ground to powder. The pulverized cells were thawed in a 30 °C water bath for 5 minutes. The lysate was centrifuged at 4 °C for 10 minutes at 20,000×*g*. The supernatant was stored at –80 °C or directly used for sucrose density gradient centrifugation to collect ribosomes-mRNA complex. 10–55% sucrose density gradient (10 mM MgCl_2_, 100 mM NH_4_Cl, 20 mM Tris, pH 8.0, 1 mM chloramphenicol and 2 mM DTT) was pro-prepared and stored overnight at 4 °C. The samples were loaded on gradients and centrifuged in an ultracentrifuge using an SW41 rotor at 35,000 rpm for 2.5 h at 4 °C. The absorption peak of ribosomes was detected at 254 nm to collect the ribosome-mRNA complex. After collection, 1% SDS was added to denature ribosome and then equal volume RNA Extraction Reagent (P1011, Solarbio Life Sciences) was added. Samples were placed on ice for 5 minutes and shaken violently for 3 minutes, then centrifuged at 4 °C for 5 minutes at 10,000 rpm. The upper water phase containing mRNA was transferred to the RNA adsorption column. The collected RNA was used for high-throughput sequencing (BGI Tech).

### Electrophoretic mobility-shift assay

The biotin‐labeled RNA (containing SBS-Up or SBS-Down) (Table S6) was synthesized by Sangon Biotech. First, the purified protein S1 was incubated with AcP (10 mM) at 37 °C for 5 h, then the reaction buffer was replaced by PBS using ultrafiltration. The concentration of AcP-acetylated S1^Ac^ was determined by BCA Protein Assay Kit (TIANGEN, PA115), then the appropriate amount of AcP-acetylated S1^Ac^ was subjected to EMSA assay. Binding reactions contained S1 or AcP-acetylated S1 (S1^Ac^), 1 µM biotin‐labeled RNA, 1× EMSA/Gel-shift binding buffer (Beyotime Biotechnology, China) were incubated at 20 °C for 30 minutes. After binding, the samples are separated on a non‐denaturating PAGE gel in ice‐bathed 0.5× Tris‐borate‐EDTA (TBE) at 170 V and bands are detected by BeyoECL Plus (Beyotime Biotechnology, China).

### *E. coli* motility assay

*E. coli* was grown on LB solid plates overnight at 37 °C. The single colonies were inoculated on LB semisolid medium containing 0.5% agar (supplement with 1 mM IPTG if necessary) by puncture method, after incubation at 37 °C for 24 h, the motility of WT::S1 and WT::S1 (K411Q/K464Q) was compared.

### Biofilm formation analysis

*E. coli* was grown overnight at 37 °C in 5 mL fresh LB liquid medium. The culture was diluted with fresh liquid LB (supplement with 1 mM IPTG if necessary) to the final concentration OD_600_~0.1 and added to 96-well microplate, three replicates were made for each sample. After incubated at 37 °C incubator for 60–84 h, OD_600_ was determined to normalize biofilm. For assay of biofilm, the cultures in 96-well plate were discarded and washed three times with 1% PBS buffer, after gently removed the residual liquid in each well and patted it dry, 200 μL methanol was added to fix the biofilm for 15 minutes. Methanol was removed and the 96-well plate was dried at room temperature. The biofilm was then stained with 200 μL, 1% crystal violet solution and the 96-well plate was shook horizontally at room temperature for 10 minutes. After dyeing, the 96-well plate was washed three times with water and dried at room temperature for 20 minutes. Finally, 200 μL 33% acetic acid was added until the biofilm was completely dissolved and OD_600_ was measured.

### Measurement of lipopolysaccharide

*E. coli* was grown overnight at 37 °C in 5 mL fresh LB liquid medium. The culture was diluted to the final concentration OD_600_~0.01 in fresh liquid LB (supplement with 1 mM IPTG if necessary) and cultured at 37 °C for 12 h at 220 rpm. The cells were collected and lyophilized repeatedly to be lysed. Concentration of lipopolysaccharide in supernatant was analyzed by lipopolysaccharide ELISA assay kit (Shanghai Enzyme-linked Biotechnology Co., Ltd.).

### Statistics and reproducibility

Statistical analyses of data other than RNC-Seq data were performed with GraphPad Prism 7 software. All results were analyzed by Student’s unpaired *t* test. *P* < 0.05 was considered statistically significant for these comparisons. *P < 0.05, **P < 0.01, ***P < 0.001. For quantification and statistical analyses, data were collected from at least three biological replicates as described in figure legends.

### Reporting summary

Further information on research design is available in the Nature Research Reporting Summary linked to this article.

## Supplementary information

Supplementary Information

Description of Additional Supplementary Files

Supplementary Data 1

Supplementary Data 2

Reporting Summary

## Data Availability

Sequences of translating mRNAs of this study are available at Mendeley Dataset, http://data.mendeley.com/datasets/jchwksxks9/draft?a=939bd9f8-a037-4645-a549-3bfe7e735630. All data generated or analyzed during this study are included in this published article (and its supplementary information files). The SBS library data are available in Supplementary Data 1. The uncropped and unedited blot/gel images are available in Supplementary Data 2.
