# Supplementary material for: Selective recruitment of stress-responsive mRNAs to ribosomes for translation by acetylated protein S1 during nutrient stress in Escherichia coli
Source: Commun Biol. 2022 Sep 1;5:892. doi: 10.1038/s42003-022-03853-4 (PMC9437053; doi:10.1038/s42003-022-03853-4)
Supplement: Supplementary file 1 — Supplementary Information [file 42003_2022_3853_MOESM1_ESM.pdf]

Supporting Information:

Selective recruitment of stress-responsive mRNAs to ribosomes for translation by acetylated protein S1 during nutrient stress in *Escherichia coli*

Bai-Qing Zhang[1], Zong-Qin Chen[1], Yu-Qi Dong[1], Di You[1]*, Ying Zhou[1]*, Bang-Ce Ye[1,2]*

[1]Laboratory of Biosystems and Microanalysis, State Key Laboratory of Bioreactor Engineering, East China University of Science and Technology, Shanghai 200237, China;

[2]Institute of Engineering Biology and Health, Collaborative Innovation Center of Yangtze River Delta Region Green Pharmaceuticals, College of Pharmaceutical Sciences, Zhejiang University of Technology, Hangzhou 310014, Zhejiang, China.

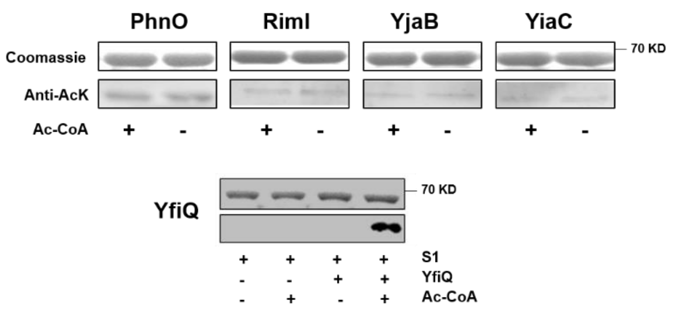

Figure S1. Five currently known acetyltransferases (RimI, YiaC, YjaB, and PhnO, YfiQ) in *E. coli* (T. D. Puente, et al., The protein acetyltransferase PatZ from *Escherichia coli* is regulated by autoacetylation-induced oligomerization. *J Biol Chem.* 290, 23077-23093, 2015) were incubated with S1, only YfiQ acetylated S1 in the presence of Ac-CoA.

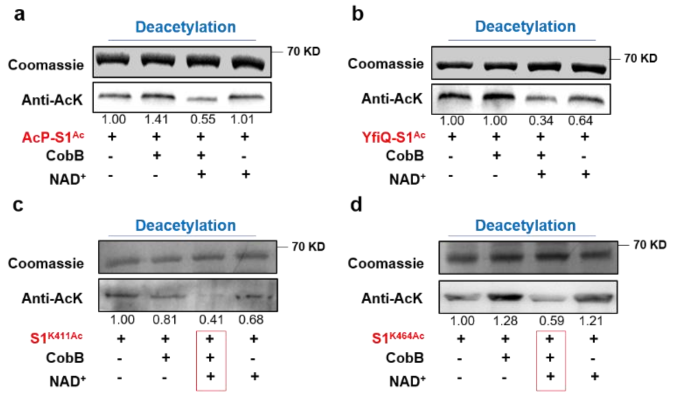

Figure S2. AcP-acetylated S1, YfiQ-acetylated S1, site-specifically lysine acetylated proteins S1^{K411Ac} and S1^{K464Ac} were partially deacetylated by *E. coli* deacetylase CobB. The band intensities were quantified by densitometry using Image J software.

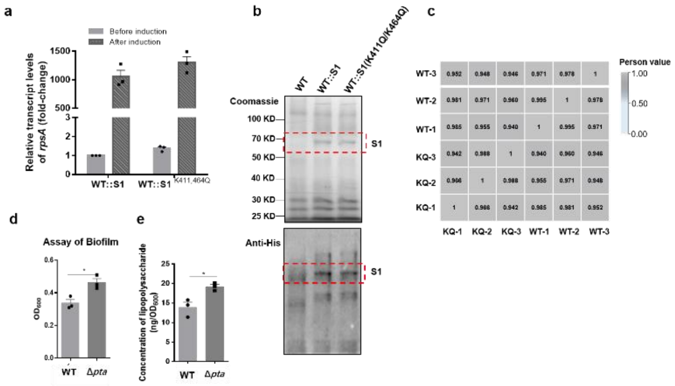

Figure S3. Preliminary validation and correlation representation in RNC-seq analysis. **a** Relative transcription of *rpsA* before or after induction in WT::S1 and WT::S1 (K411Q/K464Q). Three replicates were made for each sample. **b** His-S1 or His-S1 (K411Q/K464Q) in ribosome was detected by western-blotting assay. **c** Pearson correlation coefficients indicated a strong concordance between WT (WT-1, WT-2, WT-3) and KQ (KQ-1, KQ-2, KQ-3). **d** Biofilm assay of WT and Δ*pta* in the 96-microplate. *P < 0.05, **P < 0.01, ***P < 0.001. Three replicates were made for each sample. **e** Lipopolysaccharide of WT and Δ*pta*. *P < 0.05, **P < 0.01, ***P < 0.001. Three replicates were made for each sample.

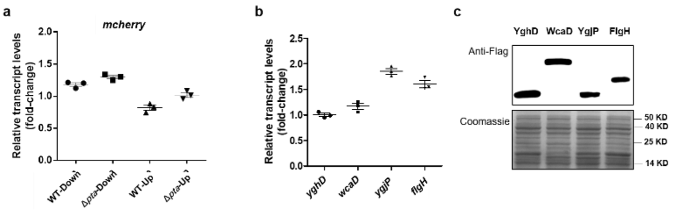

Figure S4. Relative translation levels of different test genes. **a** Relative transcription of SBS-Up or SBS-Down guiding mCherry testing element in WT or Δ*pta*. *P < 0.05, **P < 0.01, ***P < 0.001. Three replicates were made for each sample. **b** Relative transcription of up or down genes in Δ*pta*. *P < 0.05, **P < 0.01, ***P < 0.001. Three replicates were made for each sample. **c** Translation level of up or down genes in Δ*pta*. *P < 0.05, **P < 0.01, ***P < 0.001. Three replicates were made for each sample.

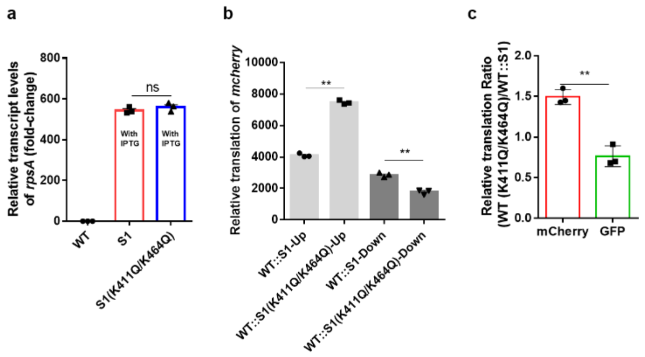

Figure S5. **a** Relative transcription of *rpsA* after induction in WT::S1 and WT::S1 (K411Q/K464Q)

possessing the fluorescent protein reporter. **b** Relative translation of mCherry mRNA with SBS-Up or with SBS-Down in WT::S1 or WT::S1 (K411Q/K464Q) strain. **c** Relative translation of SBS-Up-guiding mCherry and SBS-Down-guiding GFP in WT::S1 or WT::S1 (K411Q/K464Q) strain.

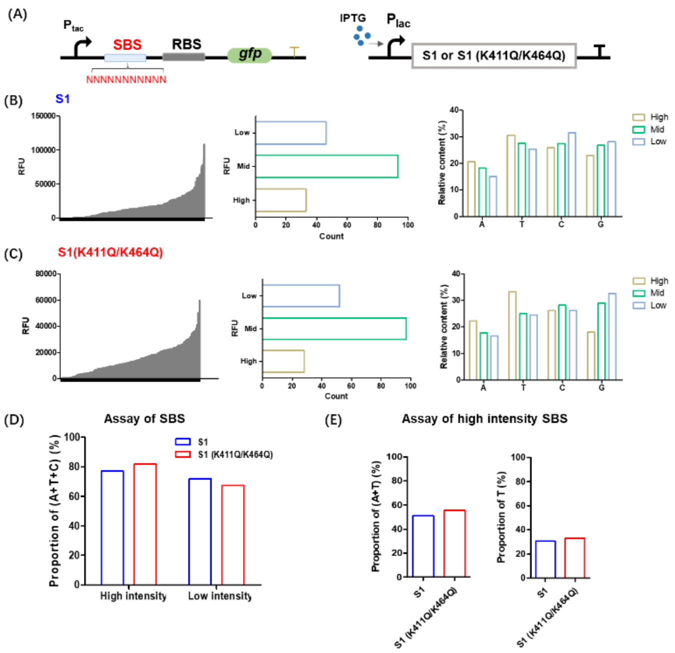

Figure S6. Design of the SBS library and identification of SBS sequences with the different translation efficiency. **a** Schematic of pKD236-P$_{tac}$-SBS-RBS-GFP library in *E. coli* BL21 possessing pProEX-S1 or pProEX-S1 (K411Q/K464Q). **b** GFP fluorescence assay, count and sequence specificity of SBSs library in S1. **c** GFP fluorescence assay, count and sequence specificity of SBSs library in S1 (K411Q/K464Q). **d** Nucleotide proportion assay of SBSs library. **e** Nucleotide proportion assay of high SBSs library.

Fig. S7 All MS/MS spectra for the identification of acetylated sites by LC/MS/MS analysis.

AcP-dependent S1 *in vitro*

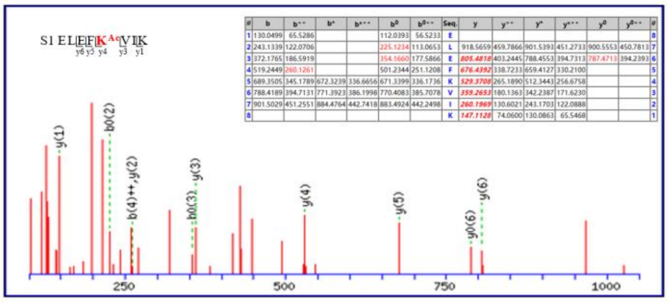

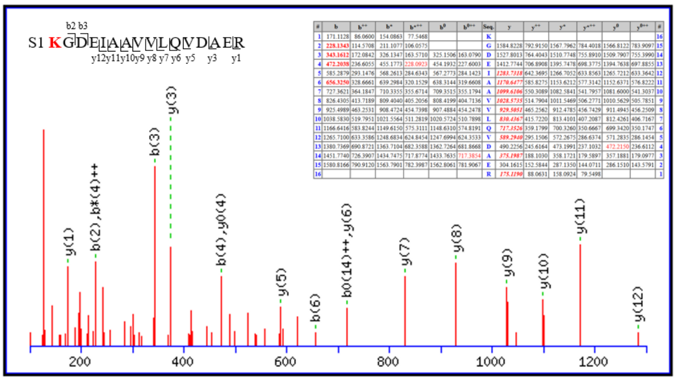

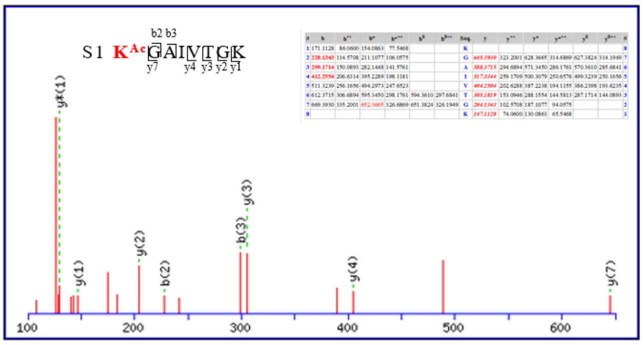

AcP-dependent S1 *in vivo*

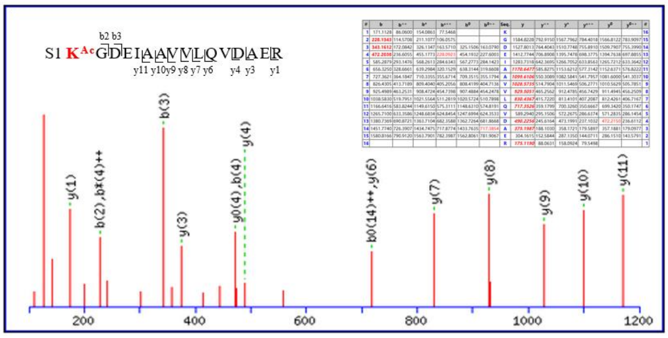

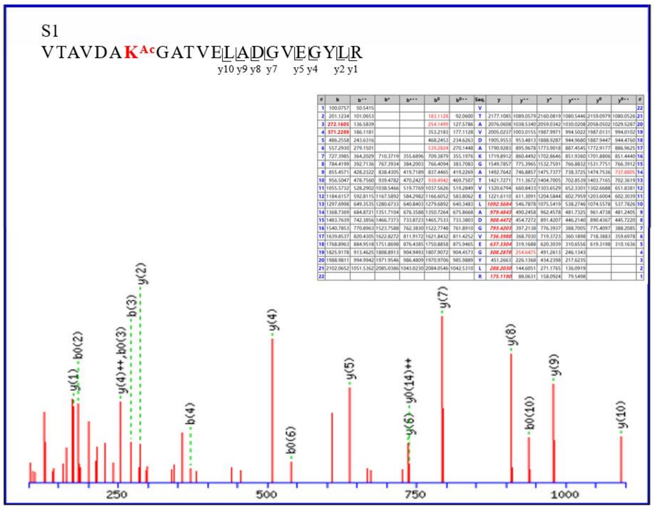

YfiQ-dependent S1 *in vitro*

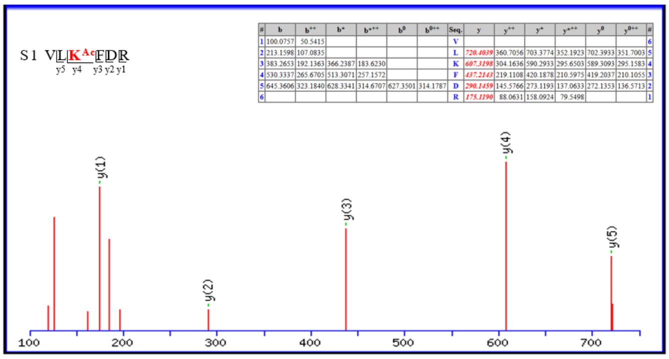

#	b	b^{++}	b^*	b^{*++}	b^0	b^{0++}	Seq.	y	y^{++}	y^*	y^{*++}	y^0	y^{0++}	#
1	100.0757	50.5415					V							6
2	213.1598	107.0835					L	*720.4039*	360.7056	703.3774	352.1923	702.3933	351.7003	5
3	383.2653	192.1363	366.2387	183.6230			K	*607.3198*	304.1636	590.2933	295.6503	589.3093	295.1583	4
4	530.3337	265.6705	513.3071	257.1572			F	*437.2143*	219.1108	420.1878	210.5975	419.2037	210.1055	3
5	645.3606	323.1840	628.3341	314.6707	627.3501	314.1787	D	*290.1459*	145.5766	273.1193	137.0633	272.1353	136.5713	2
6							R	*175.1190*	88.0631	158.0924	79.5498			1

YfiQ-dependent S1 *in vivo*

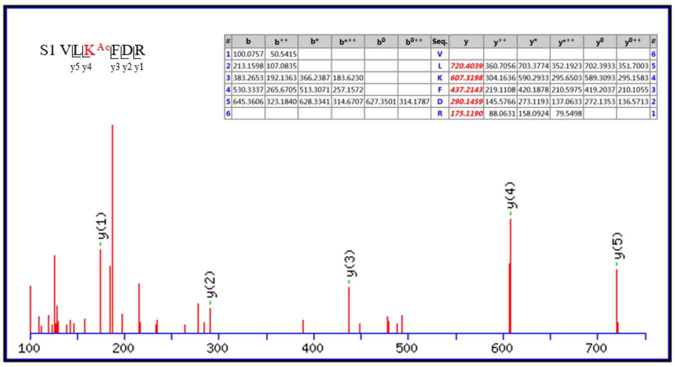

#	b	b^{++}	b^*	b^{*++}	b^0	b^{0++}	Seq.	y	y^{++}	y^*	y^{*++}	y^0	y^{0++}	#
1	100.0757	50.5415					V							6
2	213.1598	107.0835					L	*720.4039*	360.7056	703.3774	352.1923	702.3933	351.7003	5
3	383.2653	192.1363	366.2387	183.6230			K	*607.3198*	304.1636	590.2933	295.6503	589.3093	295.1583	4
4	530.3337	265.6705	513.3071	257.1572			F	*437.2143*	219.1108	420.1878	210.5975	419.2037	210.1055	3
5	645.3606	323.1840	628.3341	314.6707	627.3501	314.1787	D	*290.1459*	145.5766	273.1193	137.0633	272.1353	136.5713	2
6							R	*175.1190*	88.0631	158.0924	79.5498			1

Table S1. Secondary structual elements in native and acetylated protein S1.

Protein	α-Helix (%)	Antiparallel (%)	parallel (%)	β-turn (%)	Random coli (%)	Total sum (%)
**S1**	97.9	0	0.9	2.6	7.8	109.2
**AcP-S1^{Ac}**	99.6	0	0.1	2.7	0.3	102.7
**S1$^{K411, 464Ac}$**	99.6	0	0.1	3.0	0.2	103.0

Table S2. Acetylated sites of S1 *in vitro* and *in vivo*.

Condition	Acetylation mechanism	Acetylation sites
In vitro	AcP-dependent	K155, K411, K450, K464
	YfiQ-dependent	K247
In vivo	AcP-dependent (in Δ*yfiQ* strain)	K411, K464
	YfiQ-dependent (in Δ*pta* strain)	K247

In vitro AcP acetylation: Purified His-S1 was acetylated by AcP *in vitro*. *In vitro* YfiQ acetylation: S1 was acetylated by YfiQ and Ac-CoA *in vitro*. *In vivo* AcP acetylation: S1 was purified in Δ*yfiQ*::S1. *In vivo* YfiQ acetylation: His-S1 was purified in Δ*pta*::S1. The sequence coverage of the LC-MS/MS identification were 76% (in vitro AcP-dependent acetylation sample), 87% (in vitro YfiQ-dependent acetylation sample), 79% (in vivo AcP-dependent acetylation sample), and 73% (in vitro YfiQ-dependent acetylation sample).

Table S3. Differential expression of some genes enriched in RNC-Seq.

Gene Gene	Pathway	Log2(KQ/WT)	FC(fold change) KQ/WT
RstB	Two-component system	2.49	5.61
RstA	Two-component system	1.21	2.31
QseB	Flagella	2.30	4.92
QseC	Flagella	0.44	1.36
FliA	Flagella	1.43	2.69
FlhC	Flagella	3.19	9.13
FlhD	Flagella	1.12	2.17
DgcM	Biofilm	1.72	3.29
MlrA	Biofilm	2.07	4.20
CsgD	Biofilm	2.41	5.31
CsgB	Biofilm	3.18	9.06
GlnL	Nitrogen assimilation	1.40	2.64
GlnG	Nitrogen assimilation	1.40	2.64
NarW	Nitrogen assimilation	1.26	2.39
NarY	Nitrogen assimilation	1.19	2.28
NarZ	Nitrogen assimilation	1.87	3.66
GlrK	Amino sugar metabolism	1.38	2.60
GlrR	Amino sugar metabolism	2.35	5.10
PhoQ	Mg^{2+} transport	1.03	2.04
KdpA	K^{+} transport	2.22	4.66
KdpB	K^{+} transport	2.48	5.58
CusF	Cu^{2+} transport	3.34	10.13
CusC	Cu^{2+} transport	1.39	2.62
ZraR	Zn^{2+} transport	2.54	5.82
ZraP	Zn^{2+} transport	1.89	3.71
WaaL	Lipopolysaccharide biosynthesis	1.69	3.23
WaaQ	Lipopolysaccharide biosynthesis	2.54	5.82
WaaP	Lipopolysaccharide biosynthesis	2.11	4.32
WaaU	Lipopolysaccharide biosynthesis	1.58	2.99
WaaZ	Lipopolysaccharide biosynthesis	1.99	3.97
WaaB	Lipopolysaccharide biosynthesis	1.49	2.81
WaaJ	Lipopolysaccharide biosynthesis	1.55	2.93
WaaC	Lipopolysaccharide biosynthesis	1.68	3.20
HisB	Histidine biosynthesis	-2.52	0.17
HisC	Histidine biosynthesis	-1.95	0.26
HisA	Histidine biosynthesis	-1.17	0.44
HisD	Histidine biosynthesis	-2.96	0.13
HisF	Histidine biosynthesis	-2.72	0.15
HisH	Histidine biosynthesis	-2.31	0.20

HisI	Histidine biosynthesis	-1.28	0.41
YkgM	Ribosome	-2.53	0.17
RpsQ	Ribosome	-1.72	0.30
RpmG	Ribosome	-1.65	0.32
RpmA	Ribosome	-1.19	0.44
RplT	Ribosome	-1.23	0.43
YkgO	Ribosome	-1.02	0.49
RplL	Ribosome	-1.20	0.44
RplK	Ribosome	-1.05	0.48
RplA	Ribosome	-1.03	0.49
RpmJ	Ribosome	-1.07	0.48
RplS	Ribosome	-1.40	0.38
RpsT	Ribosome	-1.22	0.43
RpmF	Ribosome	-1.07	0.48

Table S4. Strains and plasmids used in this work.

Strain or plasmid	source or reference
**strains**	
E. coli BL21(DE3)	TransGen Biotech
E. coli MG1655 (WT)	NCBI:txid511145
WT-pProEX-S1 (WT::S1)	In this work
WT-pProEX-S1 (K411, 464Q) (WT::S1 (K411Q/K464Q))	In this work
E. coli BL21-pET-28a-S1	In this work
E. coli BL21-pTECH-MbAcK3RS/pET-28a-S1^{K411Ac}	In this work
E. coli BL21-pTECH-MbAcK3RS/pET-28a-S1^{K450Ac}	In this work
E. coli BL21-pTECH-MbAcK3RS/pET-28a-S1^{K464Ac}	In this work
E. coli BL21-pTECH-MbAcK3RS/pET-28a-S1$^{K411, 464Ac}$	In this work
Δ*pta*-pProEX-S1 (Δ*pta*::S1)	In this work
Δ*pta*	In this work
Δ*yfiQ*-pProEX-S1 (Δ*yfiQ*::S1)	In this work
WT-pUC19-SBS-Up-mcherry	In this work
WT-pUC19-SBS-Down-mcherry	In this work
Δ*pta*-pUC19-SBS-Up-mcherry	In this work
Δ*pta*-pUC19-SBS-Down-mcherry	In this work
WT::S1-pKD236-SBS-Up-mcherry	In this work
WT::S1-pKD236-Down-mcherry	In this work
WT::S1(K411, 464Q)-pKD236-Up-mcherry	In this work

WT::S1(K411, 464Q)-pKD236-Down-mcherry	In this work
WT-pUC19-SBS-Up-mcherry-SBS-Down-GFP	In this work
Δ*pta*-pUC19-SBS-Up-mcherry-SBS-Down-GFP	In this work
Δ*pta*- pUC19-*yghD*	In this work
Δ*pta*-pUC19-*wcaD*	In this work
Δ*pta*-pUC19-*ygjP*	In this work
Δ*pta*-pUC19-*flgH*	In this work
E. coli BL21-pKD236-SBS-A-GFP	In this work
E. coli BL21-(pKD236-SBS-A-GFP/pET-28a-S1-S1(K411Q/K464Q))	In this work
**plasmids**	
pET-28a	Thermo Scientific
pTECH-MbAcK3RS(IPYE)	a gift from David Liu
pUC19	Thermo Scientific
pET-28a-*rpsA*	In this work
pProEX-S1	In this work
pET-28a-*cobB*	In this work
pProEX-S1 (K411, 464Q)	In this work
puc19-SBS-Up-mcherry	In this work
puc19-SBS-Down-mcherry	In this work
pUC19-SBS-Up-mcherry-SBS-Down-GFP	In this work
pUC19-SBS-Up-mcherry-SBS-Down-GFP	In this work
pKD236-SBS-Up-mcherry	In this work
pKD236-SBS-Down-mcherry	In this work
pKD236-SBS-Up-mcherry-SBS-Down-GFP	In this work
pKD236-SBS-Up-mcherry-SBS-Down-GFP	In this work
pUC19-*yghD*	In this work
pUC19-*wcaD*	In this work
pUC19-*ygjP*	In this work
pUC19-*flgH*	In this work
pKD236	Thermo Scientific
pKD236-SBS-A-GFP	In this work
pET-28a-S1-S1 (K411Q/K464Q)	In this work

Table S5. Primers for RT-PCR and site-directed mutagenesis.

Gene	Sequence (5’-3’)

**RT-PCR**	
yifQ	GAGTCAGCGAGGACTGGAAGC
	AAACCGCAAGGTCGGGTGTAA
**Site-directed mutagenesis**	
S1	ATGACTGAATCTTTTGCTCAACTCTTTGAAGAGT
	TTACTCGCCTTTAGCTGCTTTGAAAGCTTC
$S1^{K411Q}$	TTCGTGAATACAAACAAGGCGACGAAATCGCT
	AGCGATTTCGTCGCCTTGTTTGTATTCACGAA
$S1^{K464Q}$	AGTTGACGCTCAAGGCGCAACC
	GGTTGCGCCTTGAGCGTCAACT
$S1^{K411Ac}$	GTGAATACAAATAGGGCGACGAAATCGCTGCAGTTGT
	ATTTCGTCGCCCTATTTGTATTCACGAACTGCTTCTTCGC
$S1^{K450Ac}$	CTCTGAACAAGTAGGGCGCTATCGTAACCGGTAAAGTA
	CGATAGCGCCCTACTTGTTCAGAGCAACCCAGTTGTTG
$S1^{K464Ac}$	AGTTGACGCTTAGGGCGCAACCGTAGAACTGGC
	ACGGTTGCGCCCTAAGCGTCAACTGCAGTTACTTTACC

Table S6. Sequence of biotin-labeled RNA (containing SBS-Up or SBS-Down).

	**Sequence (5'-3')**
SBS-Up-RNA	UCGGCUCGUAUAAUGUGUGGAACGCUUUCCGUAAAU AAAGGAGAUAUACC
SBS-Down-RNA	UCGGCUCGUAUAAUGUGUGGAACGCUUUUACUGUUU UAAGGAGAUAUACC
