# Supplementary material for: Selective recruitment of stress-responsive mRNAs to ribosomes for translation by acetylated protein S1 during nutrient stress in Escherichia coli
Source: Commun Biol. 2022 Sep 1;5:892. doi: 10.1038/s42003-022-03853-4 (PMC9437053; doi:10.1038/s42003-022-03853-4)
Supplement: Supplementary file 2 — Description of Additional Supplementary Files [file 42003_2022_3853_MOESM2_ESM.pdf]

Description of Additional Supplementary Files

File name: Supplementary Data 1
Description: SBS library.
