# Supplementary figures and images for: Selective recruitment of stress-responsive mRNAs to ribosomes for translation by acetylated protein S1 during nutrient stress in Escherichia coli
Source: Commun Biol. 2022 Sep 1;5:892. doi: 10.1038/s42003-022-03853-4 (PMC9437053; doi:10.1038/s42003-022-03853-4)

**Uncropped and unedited blot gel images**

Fig 1b

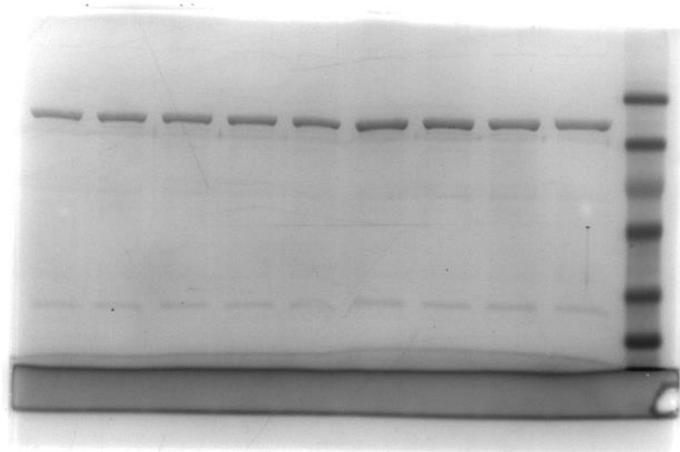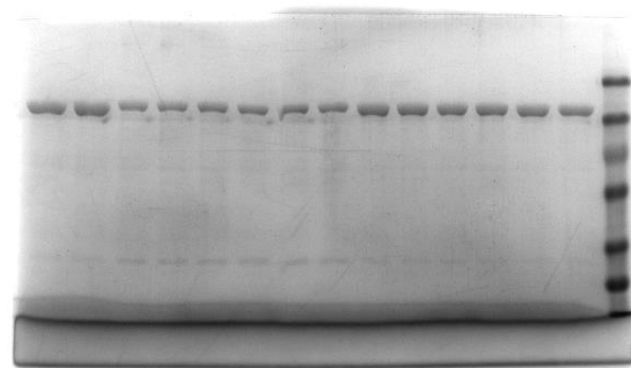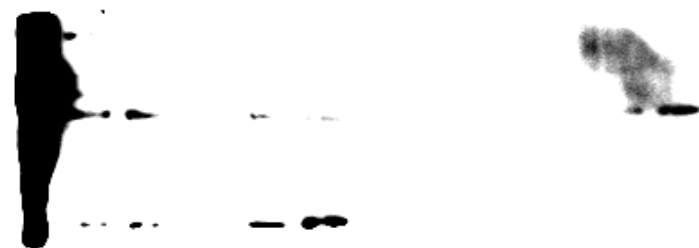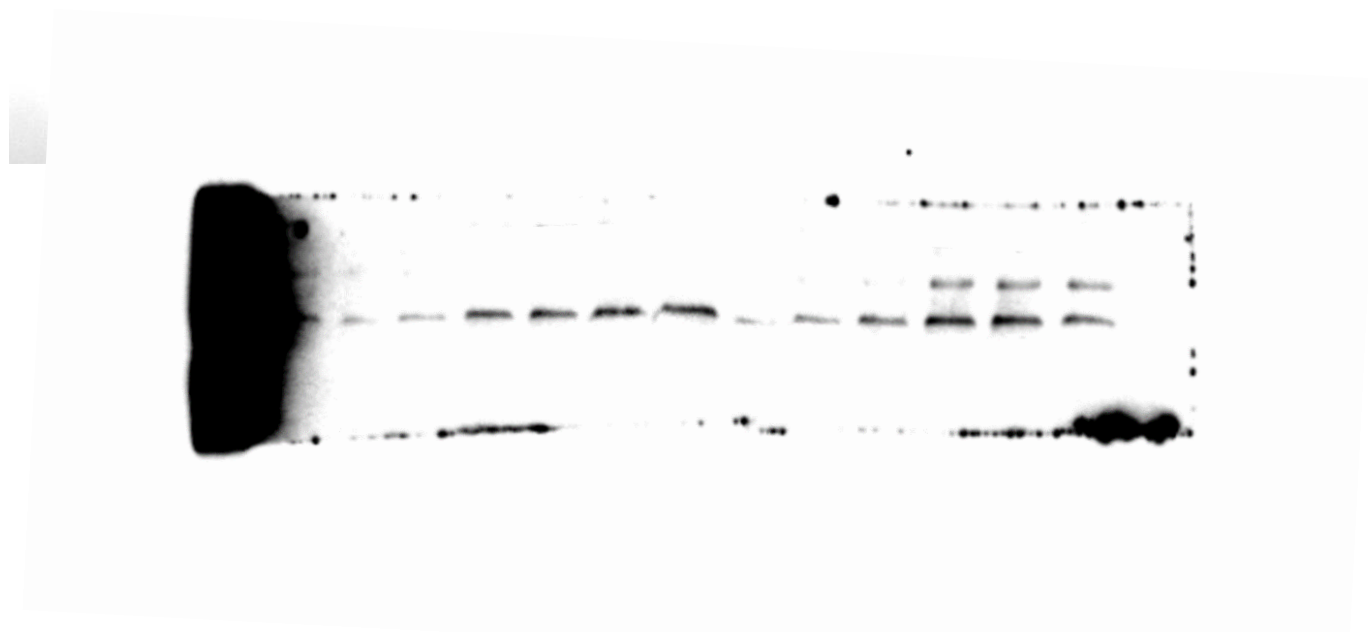

Fig 1f

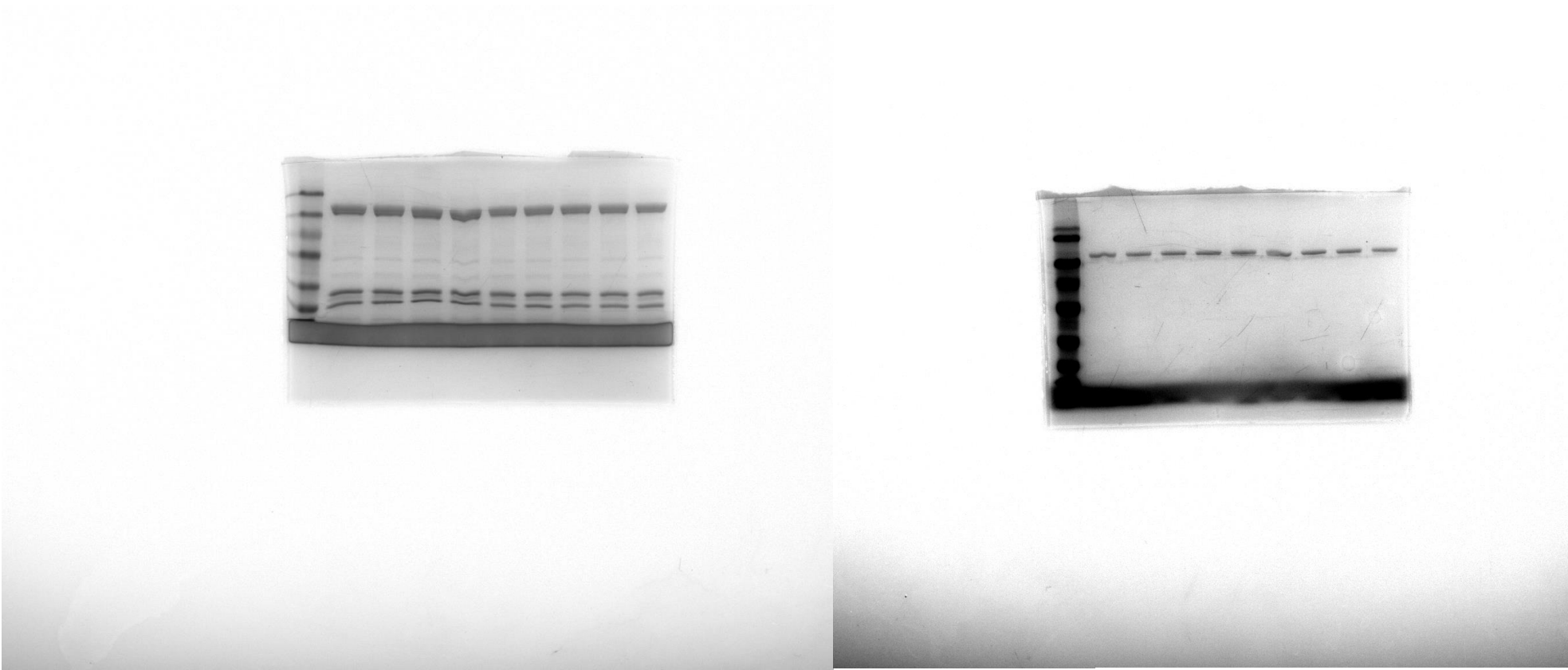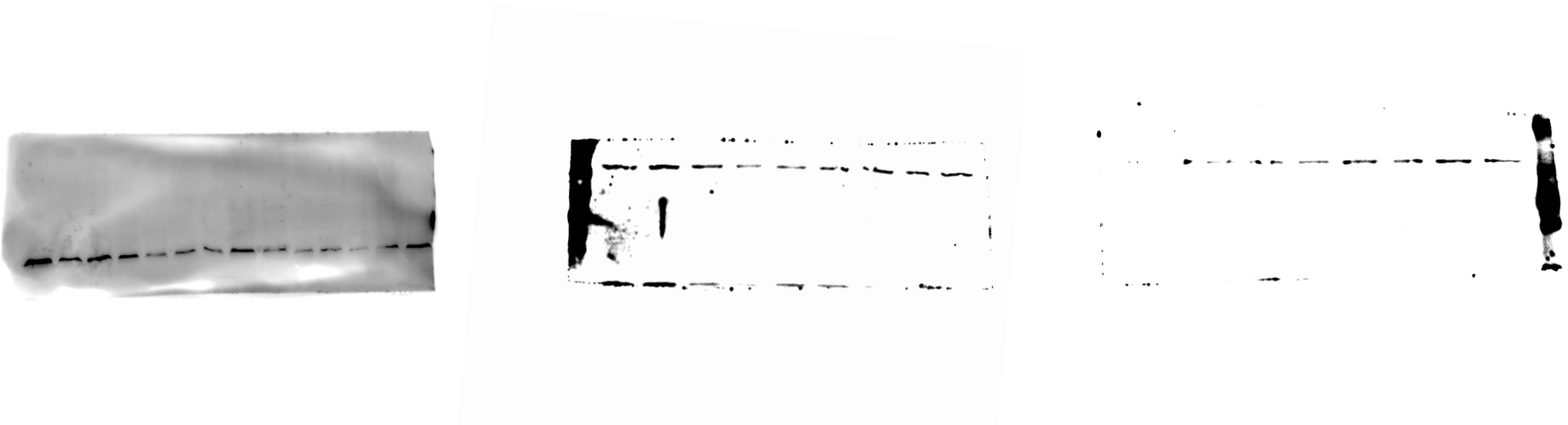

Fig 4b

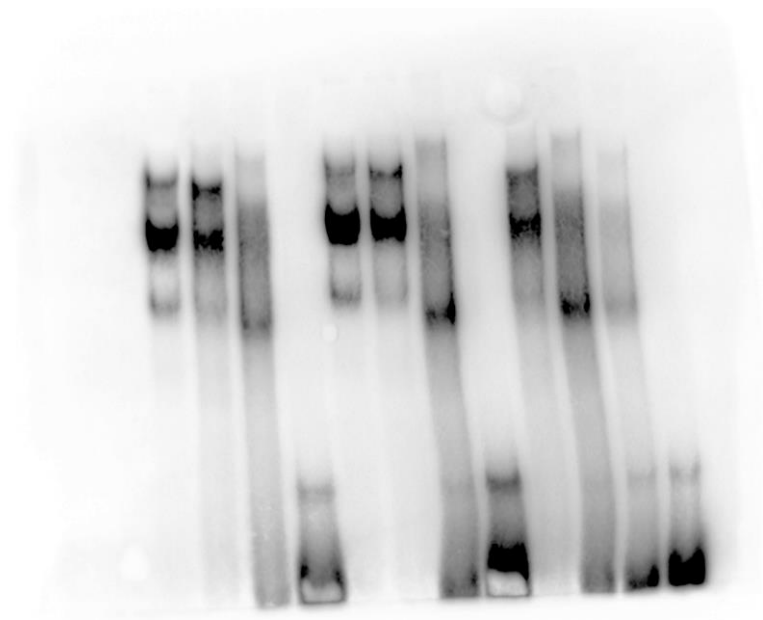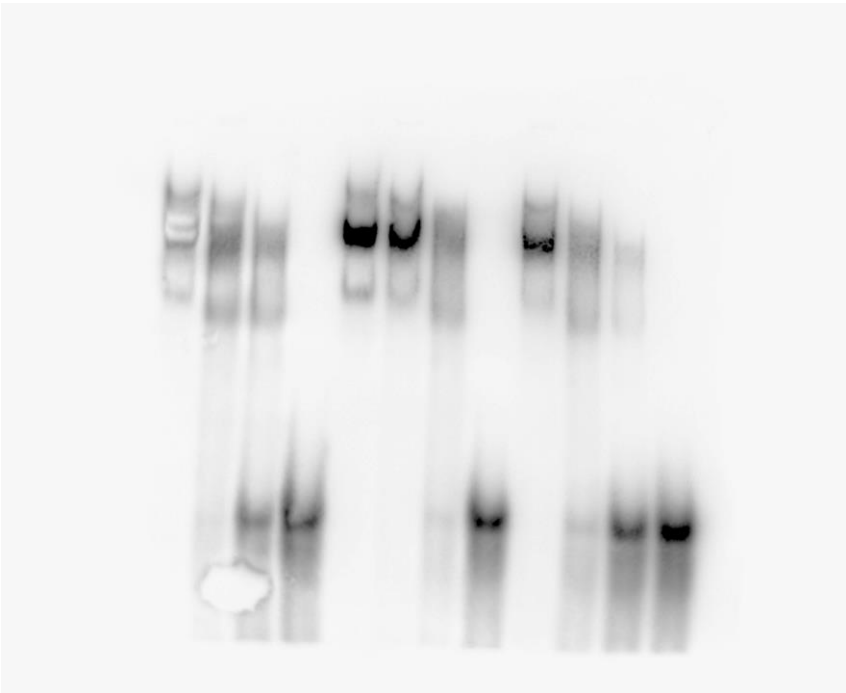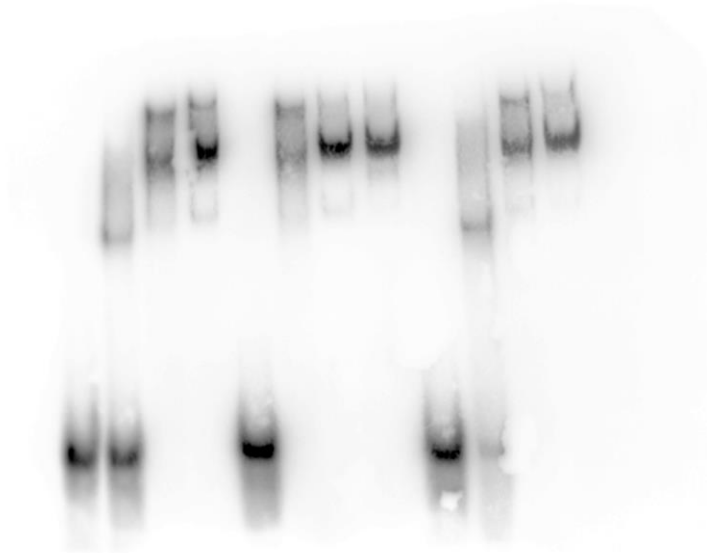

**Fig S1**

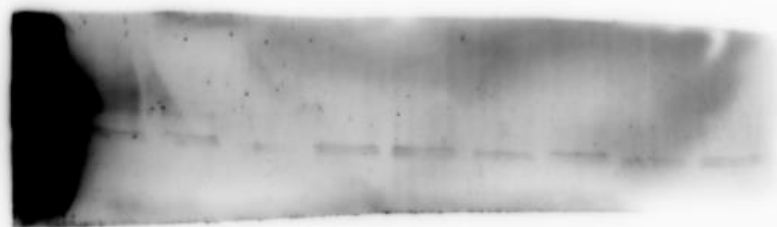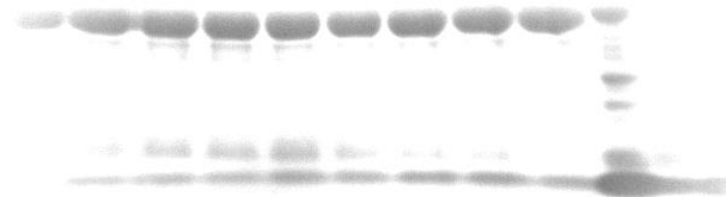

**Fig S2a, b, c**

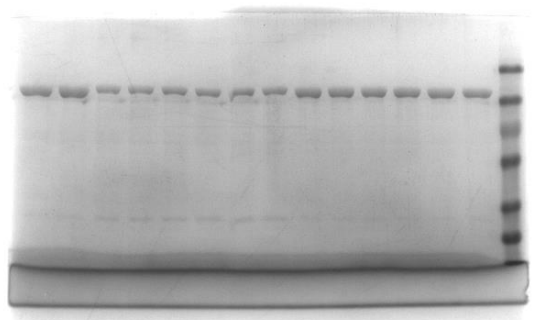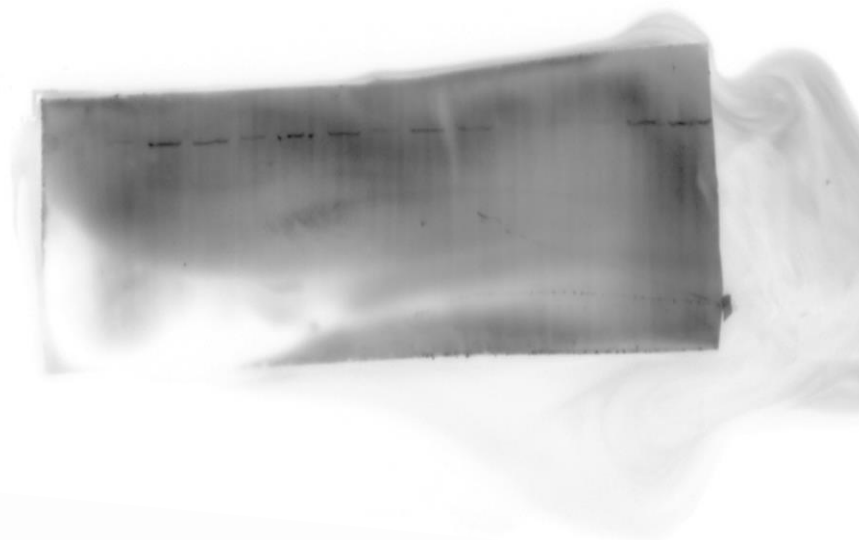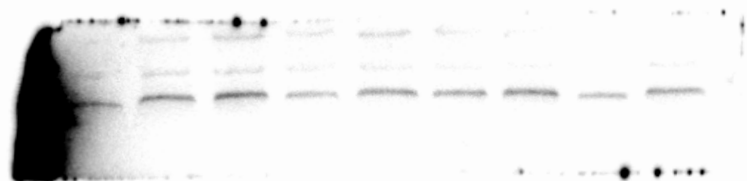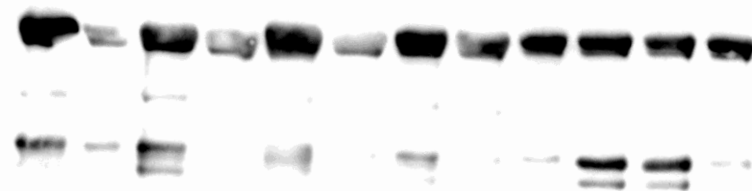

**Fig S3b**

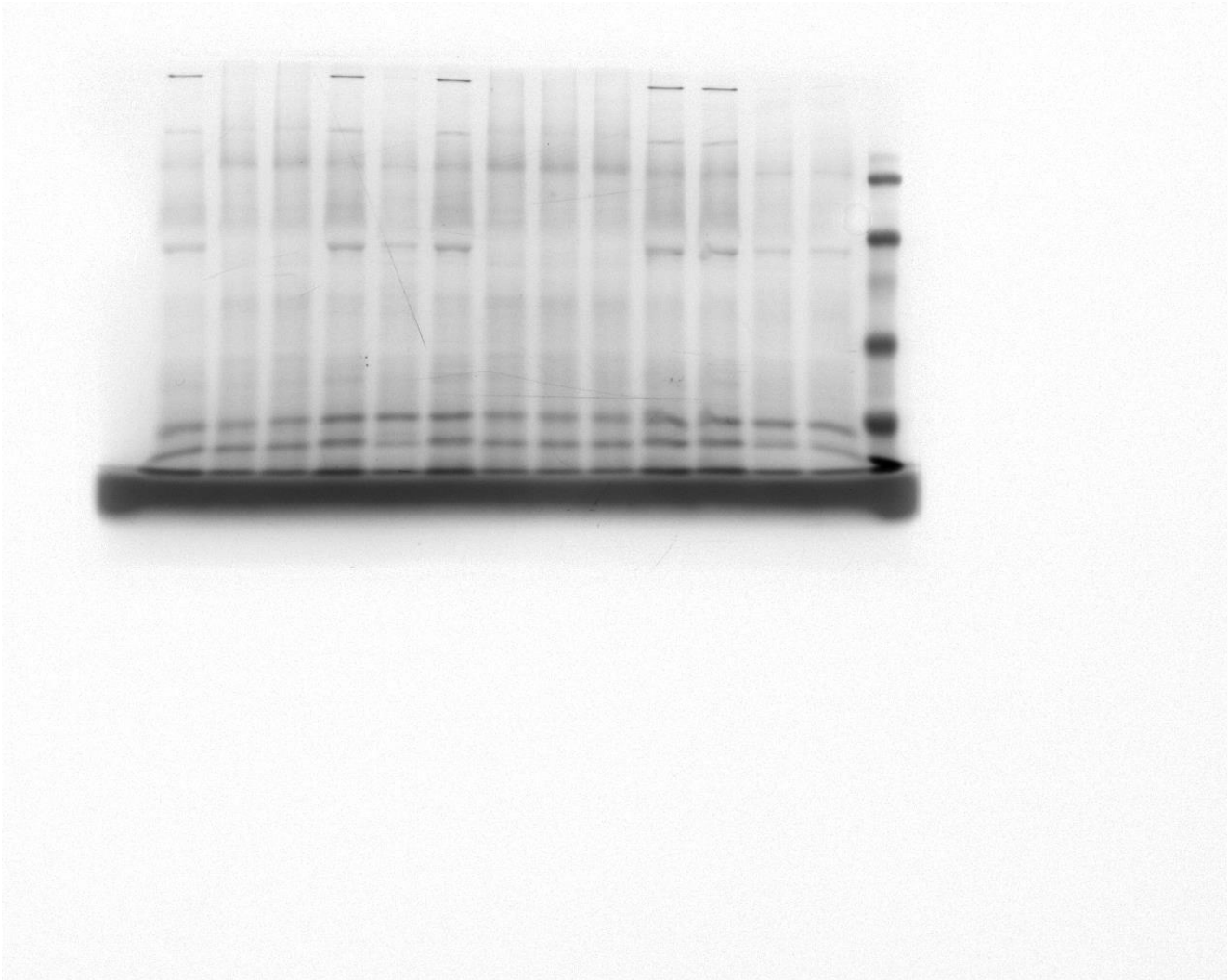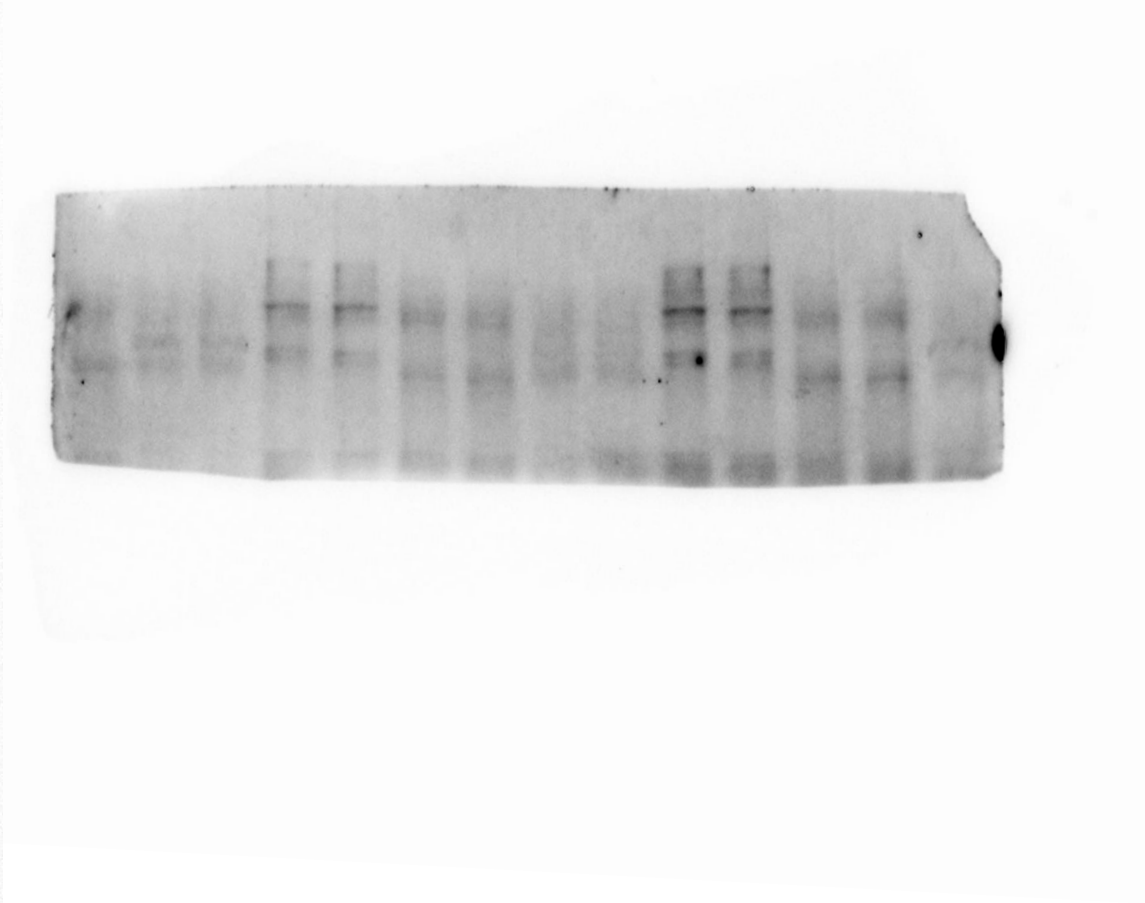

Fig S4c

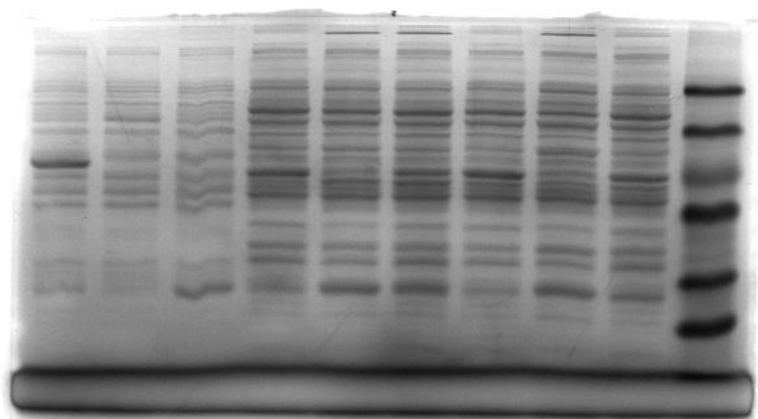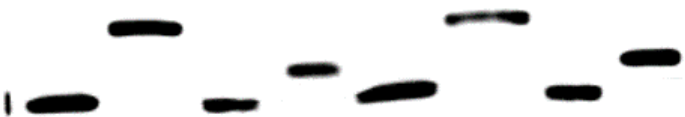

Supplement: Supplementary file 4 — Supplementary Data 2 [file 42003_2022_3853_MOESM4_ESM.pdf]
